# Simultaneous Capture and Detumble of a Resident Space Object by a Free-Flying Spacecraft-Manipulator System

**DOI:** 10.3389/frobt.2019.00014

**Published:** 2019-03-27

**Authors:** Josep Virgili-Llop, Marcello Romano

**Affiliations:** Spacecraft Robotics Laboratory, Mechanical and Aerospace Engineering Department, Naval Postgraduate School, Monterey, CA, United States

**Keywords:** spacecraft robotics, active debris removal, on-orbit servicing, computational guidance and control, convex optimization, hardware-in-the-loop experiments, sequential convex programming

## Abstract

A maneuver to capture and detumble an orbiting space object using a chaser spacecraft equipped with a robotic manipulator is presented. In the proposed maneuver, the capture and detumble objectives are integrated into a unified set of terminal constraints. Terminal constraints on the end-effector's position and velocity ensure a successful capture, and a terminal constraint on the chaser's momenta ensures a post-capture chaser-target system with zero angular momentum. The manipulator motion required to achieve a smooth, impact-free grasp is gradually stopped after capture, equalizing the momenta across all bodies, rigidly connecting the two vehicles, and completing the detumble of the newly formed chaser-target system without further actuation. To guide this maneuver, an optimization-based approach that enforces the capture and detumble terminal constraints, avoids collisions, and satisfies actuation limits is used. The solution to the guidance problem is obtained by solving a collection of convex programming problems, making the proposed guidance approach suitable for onboard implementation and real-time use. This simultaneous capture and detumble maneuver is evaluated through numerical simulations and hardware-in-the-loop experiments. Videos of the numerically simulated and experimentally demonstrated maneuvers are included as Supplementary Material.

## 1. Introduction

On-orbit servicing holds the promise to refuel, maintain, upgrade, and repair existing spacecraft as well as to actively remove orbital debris (Long et al., 2007; Ellery et al., 2008; Flores-Abad et al., 2014a). Before the servicing operations can take place, the servicing spacecraft must capture its target. Several methods to capture a space object have been proposed, but a chaser spacecraft equipped with a robotic manipulator is widely seen as a promising and versatile approach (Bonnal et al., 2013; Flores-Abad et al., 2014a; Shan et al., 2016).

The robotic capture of cooperative and attitude-stabilized spacecraft has already been demonstrated. The Space Shuttle's Remote Manipulator System (Sallaberger et al., 1997; Goodman, 2006) and the International Space Station's robotic manipulator (Stieber et al., 1997) have been used, under human control, to capture cooperative and attitude-stabilized targets. The unmanned ETS-VII (Oda, 2000; Penin et al., 2000) and Orbital Express (Ogilvie et al., 2008) missions have also demonstrated teleoperated and automated capture of cooperative and attitude-stabilized targets.

For tumbling targets, the capture maneuver is significantly more challenging, and a detumbling phase is generally required before servicing operations can start. The capture and detumble of INTELSAT VI by the STS-49 crew in 1992 (Bennett, 1993) exemplifies some of these challenges. Spinning only at a 1.44 deg/s, INTELSAT VI required multiple capture attempts before it was manually captured and detumbled by three space walking astronauts. The unexpected difficulties were later attributed to some unforeseen effects related to fuel-sloshing and contact dynamics (Bennett, 1993). Similar difficulties have been experienced in the other instances where astronauts have manually captured slow tumbling objects (Grady, 1985; Hauck and Gardner, 1985; Goodman, 2006). Despite these accomplishments, the automated capture and detumble of resident space objects remains an open challenge.

Some of the difficulties facing a capture and detumble maneuver can be attributed to its guidance complexity. The trajectory generated by the guidance algorithm must be computed in a timely manner, the obstacle avoidance and control constraints satisfied, the nonlinear multibody kinematics and dynamics dealt with, and the propellant usage minimized. Given the relevance and challenging nature of the guidance problem, the literature on it is extensive (Flores-Abad et al., 2014a; Nanjangud et al., 2018). With respect to the capture maneuver, two distinct approaches have been considered. Some authors assume that the chaser can initiate the capture maneuver at a close-enough distance, where the target's grapple fixture is within the chaser's free-floating grasping range. Only actuating the manipulator, while leaving the base-spacecraft uncontrolled, is enough to capture the target in this scenario. Minimizing the disturbance to the floating base-spacecraft is usually considered on optimization-based approaches. Examples of this approach can be found in Yoshida et al. (2006), Shah et al. (2013), Flores-Abad et al. (2014b), Flores-Abad et al. (2016), and Stolfi et al. (2017). However, a tumbling target with large appendages imposes large time-varying keep-out zone constraints, preventing the existence of a safe holding position close-enough to the target where to wait and later execute a free-floating capture maneuver. A chaser starting at a sufficiently far-away hold position and executing a free-flying roto-translation maneuver is then required. This type of maneuver has been extensively studied with a wide variety of guidance and control approaches, such as: optimal control (Aghili, 2008, 2009a; Seweryn and Banaszkiewicz, 2008; Boyarko et al., 2011), optimization-based (Jacobsen et al., 2002; Lampariello, 2010; Lampariello and Hirzinger, 2013; Gasbarri and Pisculli, 2015; MacPherson et al., 2018), model predictive control (Rybus et al., 2017), and rapidly-exploring random trees (Persson and Sharf, 2015; Rybus and Seweryn, 2015).

The detumbling maneuver has also been the focus of significant attention, with minimum time formulations (Aghili, 2008, 2009b), sliding mode and impedance control approaches (Uyama and Narumi, 2016; Zhang et al., 2017), as well as strategies that exploit the variable inertia of the resulting multibody system to reduce its kinetic energy (Rybus et al., 2014) being proposed.

Although the capture and detumble maneuvers are usually studied independently from each other, both are generally required by on-orbit servicing missions. In the proposed simultaneous capture and detumble maneuver these two maneuvers are combined. The capture and detumble objectives are encoded with a unified set of terminal constraints, forcing the chaser's control actions to simultaneously consider both objectives from the very beginning of the maneuver. Once the target is captured, the underlying conditions to detumble the newly formed chaser-target system are met, and a completely detumbled system is achieved once the manipulator's motion is gradually stopped shortly after. Combining the capture and detumble into a single maneuver can result in a quicker and, as the chaser's monenta at capture is used to detumble the RSO, a potentially more propellant-efficient proposition.

The capture objective is casted as a terminal constraint on the chaser's end-effector position and velocity, whereas the detumble objective is formulated from a momenta standpoint and casted as terminal constraint on the chaser's momenta. These two sets of terminal constraints are then fused into a unified and coherent set. The terminal constraint derived from the detumble objective is based on the premise that a detumbled system has zero angular momentum. The capture of a space object can be seen as an inelastic collision between the two vehicles, with the post-capture momenta of the combined system resulting from the combination of the individual momenta contributed by each vehicle. To obtain a post-capture system with zero angular momentum the momenta contributed by the chaser must cancel-out the target's pre-existing angular momentum.

The terminal constraint on the chaser's momenta necessarily imposes a relative velocity between both vehicles. To achieve a smooth, impact-free capture, the chaser's manipulator is actuated to obtain a zero relative velocity between the chaser's end-effector and the target's grapple fixture. This manipulator motion is maintained during, and immediately after, capture. Therefore, the newly formed chaser-target system is not instantaneously detumbled upon capture. After capture, the manipulator joint velocities are gradually slowed down to zero, equalizing the linear and angular momenta across all bodies via internal reactions (i.e., gripper contact forces and joint reactions). After the manipulator motion is stopped, the angular momentum on all bodies of the chaser-target system vanishes to zero—as dictated by the maneuver's terminal constraint. The system is then fully detumbled and no further actuation is required.

In addition to formulating the simultaneous capture and detumble maneuver, a practical guidance algorithm for it is also proposed. The proposed guidance is able to generate a full roto-translation trajectory that minimizes the control effort, meets the simultaneous capture and detumble terminal constraint, avoids collisions, and respects the chaser's control limitations. The proposed guidance algorithm is a variation from a guidance approach suitable for onboard implementation and real-time use previously developed for capture-only maneuvers (Virgili-Llop et al., 2017b,c, 2019). Here, the detumbling of the RSO is added as additional terminal conditions to this previously developed guidance approach.

The combination of the newly proposed simultaneous capture and detumble maneuver with this guidance approach has been evaluated with numerical simulations and hardware-in-the-loop experiments. The simulation results show that the guidance ability to find admissible solutions decreases as the target's pre-capture momenta increases, eventually limiting the applicability of the simultaneous capture and detumble maneuver for targets with high tumbling rates or large inertias. The experimental results on a planar air bearing table provide empirical evidence on the efficacy of the maneuver and real-time capabilities of the proposed guidance. Videos of the experiments, as well as of the simulations, are included as Supplementary Material.

The simulation and experimental results obtained with the simultaneous capture and detumble maneuvers are compared to the results obtained with our previous capture-only maneuvers (Virgili-Llop et al., 2017b,c, 2019). The simulation scenario as well as the experimental set-up are the same on both cases, allowing for a direct comparison between the two. In a capture-only maneuver the chaser's velocity at capture is constrained, getting the chaser in sync with the rotation of the target and eliminating any relative velocity between the two. If maintained, the matching velocity terminal constraint prolongs the capture conditions beyond a single instant, greatly facilitating the grasping operation. As no consideration to the post-capture momenta is given, a separate detumble maneuver is required after capture. The new contributions of this work, which are appearing for the first time in literature to the best knowledge of the authors, are: (1) the formulation and design of a simultaneous capture and detumble maneuver, and (2) the demonstration of the proposed maneuver's feasibility via extensive numerical simulations and hardware-in-the-loop experiments.

The rest of this paper is organized as follows. In section 2, the capture and detumble problem is presented, introducing the nomenclature and equations of motion. The terminal constrains enabling the simultaneous capture and detumble are derived in section 3. The convex programming based guidance is outlined in section 4 and the results of the numerical simulations are presented in section 5. The experimental setup and results are presented in section 6.

Finally, concluding remarks are drawn.

## 2. Problem and Notation

**Figure 1:**
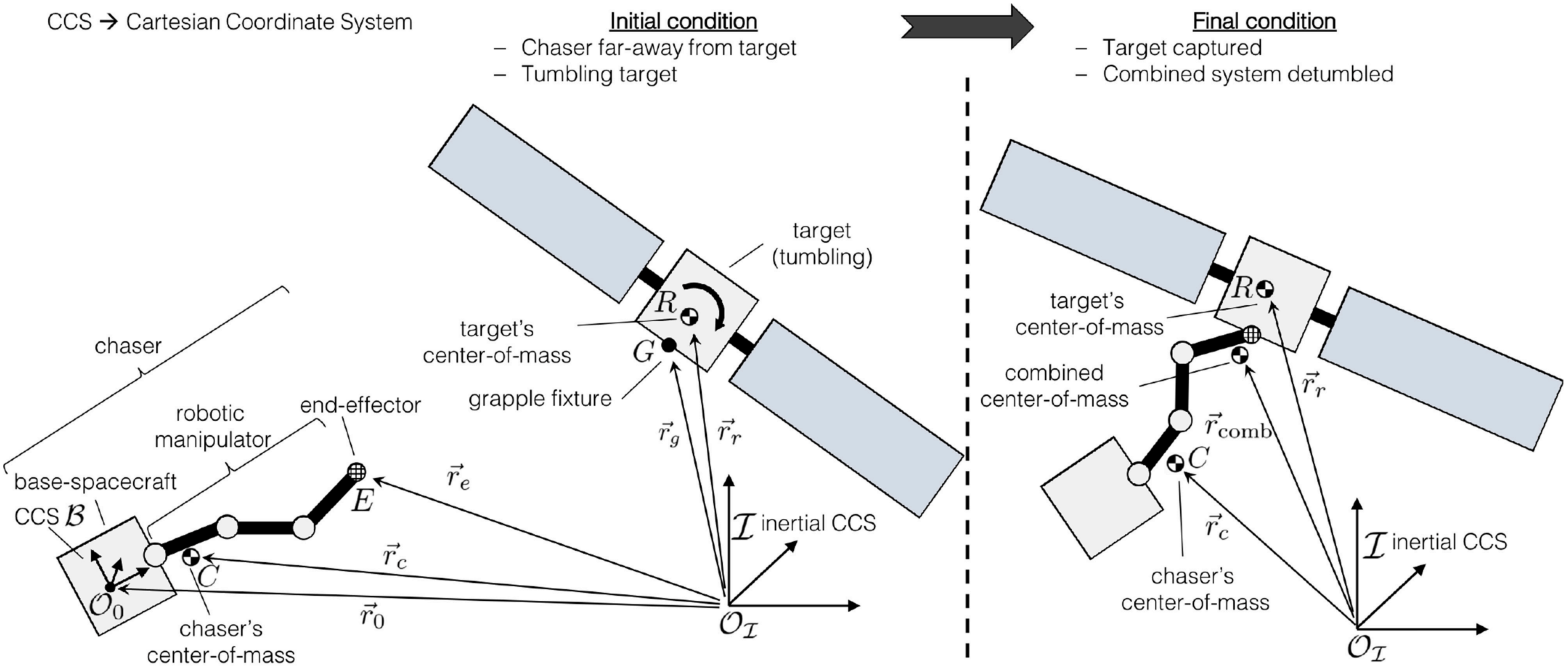
Illustration of the problem.

The capture and detumble maneuver involves a chaser spacecraft, equipped with a robotic manipulator, capturing and detumbling a Resident Space Object (RSO). A notional overview of the maneuver is shown in Figure 1.

When formulating the guidance problem, the following underlying assumptions are made Virgili-Llop et al. (2019):

**A**.1 Both the chaser and target RSO are composed of rigid bodies moving in three-dimensional space.
**A**.2 Environmental disturbances (solar radiation pressure, atmospheric drag etc.) as well as the effects of the relative orbital dynamics are neglected. This assumption is verified in section 5.2.
**A**.3 The state and inertia properties of the chaser and target RSO are known.
**A**.4 The target object to be captured has a designated grapple fixture.
**A**.5 The manipulator configuration and base-spacecraft attitude at capture, as well as the manipulator motion during the final seconds of the maneuver, *t* ≥ *t*_ps_, is pre-set and not subjected to optimization (as discussed further later). *t*_*f*_ is the capture time, *t*_ps_ marks the transition to the pre-set motion, and Δ*t*_ps_ = *t*_*f*_ − *t*_ps_ denotes the period of time when the motion is pre-set.
**A**.6 The chaser's mass remains constant during the maneuver, i.e., the amount of propellant used for the maneuver is negligibly small when compared to the chaser's mass.

### 2.1. Equations of Motion

The equations of motion of a multibody system can be written, in canonical form, as (Dubowsky and Papadopoulos, 1993):

(1) $$\begin{array}{l} \text{H}\dot{\text{u}}+\text{C}\text{u}=\tau , \end{array}$$

where ***u*** denotes the generalized velocities, **τ** the generalized forces, ***H*** the generalized inertia matrix, and ***C*** the generalized convective inertia matrix.

The generalized coordinates can be divided between those referring to the base-spacecraft (**·**)_0_ and those referring to the manipulator (**·**)_*m*_.

(2) $$\begin{array}{l} \text{u}=\left[\begin{array}{c} {\text{u}}_{0}\\ {\text{u}}_{m} \end{array}\right]\;\tau =\left[\begin{array}{c} \tau_{0}\\ \tau_{m} \end{array}\right] \end{array}$$

The number of degrees-of-freedom of the manipulator is denoted by *n*_DoF_, implying that ${\text{u}}_{m},\tau_{m}\in {\mathbb{R}}^{n_{\text{DoF}}}$ and $\text{H},\text{C}\in {\mathbb{R}}^{\left(6+n_{\text{DoF}}\right)\times \left(6+n_{\text{DoF}}\right)}$. The angular or linear displacements of the manipulator joints are denoted by $\theta_{m}\in {\mathbb{R}}^{n_{\text{DoF}}}$ with ${\dot{\theta }}_{m}={\text{u}}_{m}$.

Invoking assumption *A*.2, a Cartesian Coordinate System (CCS) can be considered as inertial if its origin is chosen as a reference orbiting point and if the orientation of its axes is kept inertially fixed (the relative orbital dynamics are neglected). Without lacking generality, all vectors—unless explicitly specified—are projected into this inertial CCS I, so in general, for a vector $\vec{a}$, the 3 × 1 column matrix containing the components of the projection of $\vec{a}$ into I is denoted by ${\text{a}}^{\left\{I\right\}}\in {\mathbb{R}}^{3}$, or simply ***a***. In addition, all kinematic quantities are—unless otherwise specified—referring to the origin of the inertial frame.

The base-spacecraft generalized velocities, ${\text{u}}_{0}\in {\mathbb{R}}^{6}$, contain the base-spacecraft's linear (${\dot{\text{r}}}_{0}\in {\mathbb{R}}^{3}$) and angular ($\omega_{0}\in {\mathbb{R}}^{3}$) velocities. Equivalently, the base-spacecraft generalized forces, $\tau_{0}\in {\mathbb{R}}^{6}$, contain the resulting force (${\text{f}}_{0}\in {\mathbb{R}}^{3}$) and torque (${\text{n}}_{0}\in {\mathbb{R}}^{3}$) applied to the base-spacecraft center-of-mass.

(3) $$\begin{array}{l} {\text{u}}_{0}=\left[\begin{array}{c} {\dot{\text{r}}}_{0}\\ \omega_{0} \end{array}\right]\;\tau_{0}=\left[\begin{array}{c} {\text{f}}_{0}\\ {\text{n}}_{0} \end{array}\right] \end{array}$$

The contributions from the base-spacecraft, manipulator, and the base-manipulator coupling can be exposed within the inertia matrices and equations of motion:

(4) $$\begin{array}{l} \left[\begin{array}{cc} {\text{H}}_{0} & {\text{H}}_{0m}\\ {\text{H}}_{0m}^{T} & {\text{H}}_{m} \end{array}\right]\left[\begin{array}{c} {\dot{\text{u}}}_{0}\\ {\dot{\text{u}}}_{m} \end{array}\right]+\left[\begin{array}{cc} {\text{C}}_{0} & {\text{C}}_{0m}\\ {\text{C}}_{m0} & {\text{C}}_{m} \end{array}\right]\left[\begin{array}{c} {\text{u}}_{0}\\ {\text{u}}_{m} \end{array}\right]=\left[\begin{array}{c} \tau_{0}\\ \tau_{m} \end{array}\right] \end{array}$$

Finally, let ${\text{r}}_{0}\in {\mathbb{R}}^{3}$ denote the base-spacecraft position and ${\text{q}}_{0}\in S^{3}$ denote a unit quaternion (***q***_0_∈{ℍ∣‖***q***_0_‖ = 1}), representing the orientation of the base-spacecraft CCS B with respect to the inertial CCS I. The differential kinematics of the attitude quaternion can be written as:

(5) $$\begin{array}{l} {\dot{\text{q}}}_{0}=\frac{1}{2}\omega_{0}⊗{\text{q}}_{0} \end{array}$$

with ⊗ denoting the quaternion product and assuming that **ω**_0_ is promoted to a pure quaternion (i.e., with zero scalar part) (Wie, 2008).

## 3. Simultaneous Capture and Detumble Maneuver

The post-capture rotational state of the combined chaser-target system is governed by the resulting angular momentum of the combined system. When the chaser captures the target RSO the linear and angular momenta of both vehicles are combined. Regulating the momenta of the chaser at capture allows to control the post-capture angular momentum of the combined chaser-target system. In the proposed simultaneous capture and detumble maneuver the chaser's momenta at capture are constrained in order to achieve a zero post-capture angular momentum on the combined chaser-target system.

The linear ${\text{p}}_{c}\in {\mathbb{R}}^{3}$ and angular ${\text{h}}_{c}\in {\mathbb{R}}^{3}$ momenta of the chaser are obtained, using the generalized velocities and inertia, as follows (Yoshida and Umetani, 1993):

(6) $$\begin{array}{l} {\text{H}}_{0}{\text{u}}_{0}+{\text{H}}_{0m}{\text{u}}_{m}=\left[\begin{array}{c} {\text{p}}_{c}\\ {\text{h}}_{c} \end{array}\right] \end{array}$$

The chaser's linear momentum can also be expressed as:

(7) $$\begin{array}{l} {\text{p}}_{c}=m_{c}{\dot{\text{r}}}_{c} \end{array}$$

where *m*_*c*_ denotes the chaser's total mass and ***r***_*c*_ the position of the chaser's center-of-mass (see Figure 1).

Furthermore, the linear and angular momenta of the target RSO can be written as:

(8) $$\begin{array}{l} {\text{p}}_{r}=m_{r}{\dot{\text{r}}}_{r} \end{array}$$

(9) $$\begin{array}{l} {\text{h}}_{r}={\text{I}}_{r}\omega_{r} \end{array}$$

with *m*_*r*_, ***I***_*r*_, and **ω**_*r*_ denoting the mass, inertia matrix, and angular velocity with respect to the inertial frame of the target RSO.

After the chaser captures the target RSO, as shown in Figure 1, the momenta of the newly formed chaser-target system becomes a combination of the momenta of the chaser and target RSO. The combined linear momentum, ***p***_comb_, is simply the sum of the individual linear momenta:

(10) $$\begin{array}{l} {\text{p}}_{\text{comb}}={\text{p}}_{c}+{\text{p}}_{r} \end{array}$$

The center-of-mass position of the newly formed chaser-target system is:

(11) $$\begin{array}{l} {\text{r}}_{\text{comb}}=\frac{m_{c}{\text{r}}_{c}+m_{r}{\text{r}}_{r}}{m_{c}+m_{r}} \end{array}$$

The angular momentum of the combined system, ***h***_comb_, using as pole the combined center-of-mass, is obtained as follows:

(12) $$\begin{array}{l} {\text{h}}_{\text{comb}}={\text{h}}_{c}+{\left({\text{r}}_{c}-{\text{r}}_{\text{comb}}\right)}^{\times }{\text{p}}_{c}+{\text{h}}_{r}+{\left({\text{r}}_{r}-{\text{r}}_{\text{comb}}\right)}^{\times }{\text{p}}_{r} \end{array}$$

with the (·)^×^ operator representing the matricial equivalent of the vector cross product,

(13) $$\begin{array}{l} {\text{a}}^{\times }=\left[\begin{array}{ccc} 0 & -a_{3} & a_{2}\\ a_{3} & 0 & -a_{1}\\ -a_{2} & a_{1} & 0 \end{array}\right] \end{array}$$

Combining Equation (12) with Equation (11) yields:

(14) $$\begin{array}{l} {\text{h}}_{\text{comb}}={\text{h}}_{r}+{\text{h}}_{c}+\frac{m_{c}m_{r}}{m_{c}+m_{r}}{\left({\text{r}}_{c}-{\text{r}}_{r}\right)}^{\times }\left({\dot{\text{r}}}_{c}-{\dot{\text{r}}}_{r}\right) \end{array}$$

The target RSO to be captured is assumed to be uncontrolled and subjected to negligible perturbations, thus with constant linear and angular momenta. Additionally, the tumbling RSO center-of-mass is assumed to be initially at rest with respect to the inertial frame I. Thus, before the capture occurs, the RSO's linear velocity and momentum are zero.

(15) $$\begin{array}{l} {\dot{\text{r}}}_{r}=0\;{\text{p}}_{r}=0\;\text{for }t<t_{f} \end{array}$$

These assumptions allow to simplify the expressions for the combined momenta as:

(16) $$\begin{array}{l} {\text{p}}_{\text{comb}}={\text{p}}_{c} \end{array}$$

(17) $$\begin{array}{l} {\text{h}}_{\text{comb}}={\text{h}}_{r}+{\text{h}}_{c}+\frac{m_{c}m_{r}}{m_{c}+m_{r}}{\left({\text{r}}_{c}-{\text{r}}_{r}\right)}^{\times }{\dot{\text{r}}}_{c} \end{array}$$

### 3.1. Terminal Constraint for a Simultaneous Capture and Detumble Maneuver

#### 3.1.1. Constraint on the Chaser's Momenta

In the proposed simultaneous capture and detumble maneuver the goal is to achieve a zero post-capture angular momentum:

(18) $$\begin{array}{l} {\text{h}}_{\text{comb}}=0 \end{array}$$

which imposes the following terminal constraint on the chaser's angular momentum, ***h***_*c*_, and linear velocity, ${\dot{\text{r}}}_{c}$:

(19) $$\begin{array}{l} {\text{h}}_{c}=-{\text{h}}_{r}-\frac{m_{c}m_{r}}{m_{c}+m_{r}}{\left({\text{r}}_{c}-{\text{r}}_{r}\right)}^{\times }{\dot{\text{r}}}_{c} \end{array}$$

This underlying terminal constraint can be casted in terms of generalized velocities ***u*** and incorporated in an optimization-based guidance approach. To do so, let's assume that the chaser's position and configuration at capture, ***r***_*c*_, ***q***_0_, **θ**_*m*_, are fixed (how to set the terminal attitude is discussed in section 4.3):

(20) $$\begin{array}{l} \theta_{m}\left(t_{f}\right)=\theta_{m}^{f} \end{array}$$

(21) $$\begin{array}{l} {\text{r}}_{c}\left(t_{f}\right)={\text{r}}_{c}^{f} \end{array}$$

(22) $$\begin{array}{l} {\text{q}}_{0}\left(t_{f}\right)={\text{q}}_{0}^{f} \end{array}$$

Therefore, the chaser velocities at capture, ***u***(*t*_*f*_), are the only remaining variables left to impose the zero angular momentum constraint defined in Equation (19). The linear velocity at capture, ${\dot{\text{r}}}_{c}\left(t_{f}\right)$, can be constrained as follows in order to neutralize the RSO's angular momentum:

(23) $$\begin{array}{l} {\dot{\text{r}}}_{c}\left(t_{f}\right)={\dot{\text{r}}}_{c}^{f}=\frac{m_{c}+m_{r}}{m_{c}m_{r}}{\left({\left({\text{r}}_{r}^{f}-{\text{r}}_{c}^{f}\right)}^{\times }\right)}^{+}{\text{h}}_{r} \end{array}$$

with the (·)^+^ operator denoting the Moore-Penrose pseudoinverse.

As ${\text{r}}_{r}^{f}-{\text{r}}_{c}^{f}$ is, in general, not perpendicular to ***h***_*r*_, there is some leftover RSO's angular momentum that needs to be neutralized by the contributing chaser's angular momentum at capture ***h***_*c*_(*t*_*f*_).

(24) $$\begin{array}{l} {\text{h}}_{c}\left(t_{f}\right)={\text{h}}_{c}^{f}=-{\text{h}}_{r}-\frac{m_{c}m_{r}}{m_{c}+m_{r}}{\left({\text{r}}_{c}^{f}-{\text{r}}_{r}^{f}\right)}^{\times }{\dot{\text{r}}}_{c}^{f} \end{array}$$

In conclusion, in order to achieve a simultaneous capture and detumble, the chaser's linear velocity and angular momentum at capture need to obey both Equations (23) and (24).

#### 3.1.2. Constraint on the Chaser's Attitude and Manipulator's Motion

In addition to detumble the target, a smooth, impact-free capture is desired. In order to achieve it, a zero relative velocity between the chaser's end-effector and target's grappling fixture is enforced, ensuring that the gripper can gently capture the grappling fixture without incurring in velocity discontinuities that could generate high contact forces. This condition implies that the position and velocity of the chaser's manipulator end-effector *E* must match the position and velocity of the target's grapple fixture *G*.

(25) $$\begin{array}{l} {\text{r}}_{e}\left(t_{f}\right)={\text{r}}_{g}\left(t_{f}\right) \end{array}$$

(26) $$\begin{array}{l} {\dot{\text{r}}}_{e}\left(t_{f}\right)={\dot{\text{r}}}_{g}\left(t_{f}\right) \end{array}$$

(27) $$\begin{array}{l} \omega_{e}\left(t_{f}\right)=\omega_{r}\left(t_{f}\right) \end{array}$$

where:

(28) $$\begin{array}{l} {\dot{\text{r}}}_{g}=\omega_{r}^{\times }\left({\text{r}}_{g}-{\text{r}}_{r}\right) \end{array}$$

In practice, this zero relative velocity condition is achieved by actuating the chaser's manipulator, such that the end-effector velocity matches the grapple fixture velocity. This manipulator motion is also exploited to absorb the relative velocity between the chaser and target (see Equation 23).

Let's now map the operational-space velocities of the end-effector, ${\dot{\text{r}}}_{e}$, to joint-space velocities, ***u***_*m*_, while imposing the required base-spacecraft rotation, **ω**_0_, to maintain the prescribed chaser's angular momentum, ${\text{h}}_{c}^{f}$, defined in Equation (24). The linear velocity of the end-effector, relative to the center-of-mass, ${\dot{\text{r}}}_{e,c}$, is:

(29) $$\begin{array}{l} {\dot{\text{r}}}_{e,c}\left(t_{f}\right)={\dot{\text{r}}}_{e}\left(t_{f}\right)-{\dot{\text{r}}}_{c}\left(t_{f}\right)={\dot{\text{r}}}_{g}\left(t_{f}\right)-{\dot{\text{r}}}_{c}\left(t_{f}\right) \end{array}$$

The twist of the end-effector, ***t***_*e,c*_, encapsulating the angular and relative linear velocities, can be written as follows:

(30) $$\begin{array}{l} {\text{t}}_{e,c}=\left[\begin{array}{c} {\dot{\text{r}}}_{e,c}\\ \omega_{e} \end{array}\right]=\left[\begin{array}{c} {\dot{\text{r}}}_{g}-{\dot{\text{r}}}_{c}\\ \omega_{r} \end{array}\right] \end{array}$$

The end-effector Jacobians ***J***_0_ and ***J***_*m*_ map the joint-space velocities into operational-space velocities:

(31) $$\begin{array}{l} {\text{t}}_{e,c}={\text{J}}_{0}\left[\begin{array}{c} {\dot{\text{r}}}_{0,c}\\ \omega_{0} \end{array}\right]+{\text{J}}_{m}{\text{u}}_{m} \end{array}$$

The inverse differential kinematics mapping can then be obtained:

(32) $$\begin{array}{l} {\text{u}}_{m}={\text{J}}_{m}^{+}\left({\text{t}}_{e,c}-{\text{J}}_{0}\left[\begin{array}{c} {\dot{\text{r}}}_{0,c}\\ \omega_{0} \end{array}\right]\right) \end{array}$$

The relative linear momentum of the chaser, with respect to its center-of-mass is, by definition, zero:

(33) $$\begin{array}{l} {\text{p}}_{c,c}=0 \end{array}$$

The chaser's momenta, using the relative linear momentum, can then be written as follows:

(34) $$\begin{array}{l} {\text{H}}_{0}\left[\begin{array}{c} {\dot{\text{r}}}_{0,c}\\ \omega_{0} \end{array}\right]+{\text{H}}_{0m}{\text{u}}_{m}=\left[\begin{array}{c} 0\\ {\text{h}}_{c} \end{array}\right] \end{array}$$

By combining the velocity mapping and momenta equation the following expression is obtained:

(35) $$\begin{array}{l} \left[\begin{array}{c} {\dot{\text{r}}}_{0,c}\\ \omega_{0} \end{array}\right]={\left({\text{H}}_{0}-{\text{H}}_{0m}{\text{J}}_{m}^{+}{\text{J}}_{0}\right)}^{-1}\left(\left[\begin{array}{c} 0\\ {\text{h}}_{c} \end{array}\right]-{\text{H}}_{0m}{\text{J}}_{m}^{+}{\text{t}}_{e,c}\right) \end{array}$$

#### 3.1.3. Combined Terminal Constraints

The terminal constraints for the simultaneous capture and detumble maneuver are now fully determined:

$$\begin{array}{l} \;\theta_{m}^{f},r_{c}^{f},q_{0}^{f}\to \text{assumed known},\text{see section}\;4.3\\ \;{\dot{r}}_{c}^{f}\to \text{obtained using Equation }(23)\\ \;\omega_{0}^{\text{f}}\to \text{obtained using Equation}\;(35),\text{with }h_{c}^{f}\\ \text{ given by Equation}\;(24)\\ \;{\dot{r}}_{0,c}^{f}\to \text{ obtained using Equation }\;(35)\\ \;u_{m}^{f}\to \text{ obtained using Equation }\;(32) \end{array}$$

(36) $$\begin{array}{l} {\dot{\text{r}}}_{0}^{f}={\dot{\text{r}}}_{c}^{f}+{\dot{\text{r}}}_{0,c}^{f} \end{array}$$

(37) $$\begin{array}{l} {\text{r}}_{0}^{f}={\text{r}}_{c}^{f}+{\text{r}}_{0,c}^{f} \end{array}$$

(38) $$\begin{array}{lll} {\text{r}}_{0,c}^{f} & \to & \text{obtained via direct kinematics }{\text{r}}_{0,c}^{f}=f\left(\theta_{m}^{f},{\text{q}}_{0}^{f}\right) \end{array}$$

### 3.2. Pre-set Manipulator Motion

During the last phase of the capture maneuver, *t* ∈ [*t*_ps_, *t*_*f*_], the manipulator motion is pre-set (see assumption **A**.5). The need to pre-set the manipulator motion is required by the guidance algorithm in order to handle the obstacle avoidance considerations. More details are offered in section 4 and in Virgili-Llop et al. (2019).

The manipulator configuration at capture, $\theta_{m}^{f}$, is assumed to be also pre-set, and the manipulator velocity at capture, ${\text{u}}_{m}^{f}$, is constrained by the terminal constraint presented in section 3.1.3. In order to define the manipulator's motion during the pre-set period a manipulator velocity profile is defined:

(39) $$\begin{array}{l} {\text{u}}_{m}\left(t\right)={\text{u}}_{m}^{\text{ps}}\left(t\right)\text{for}t\in \left[t_{\text{ps}},t_{f}\right] \end{array}$$

Any arbitrary velocity profile is allowed, but to illustrate the method and simplify the guidance, let's assume that the manipulator ramps-up, from a zero velocity ***u***_*m*_(*t*) = **0** at *t*_ps_ to its prescribed velocity at capture, ${\text{u}}_{m}^{f}$, with a constant acceleration:

(40) $$\begin{array}{l} {\dot{\text{u}}}_{m}^{\text{ps}}=\frac{{\text{u}}_{m}^{f}}{\Delta t_{\text{ps}}} \end{array}$$

Integrating back in time allows to obtain **θ**_*m*_(*t*_ps_) during the pre-set time period Δ*t*_ps_.

(41) $$\begin{array}{l} \theta_{m}\left(t\right)=\theta_{m}^{\text{ps}}\left(t\right)\text{for}t\in \left[t_{\text{ps}},t_{f}\right] \end{array}$$

If the manipulator velocity at capture is high, Δ*t*_*ps*_ can be shortened to ensure that the joint displacements, **θ**_*m*_, remain within limits.

**Figure 2:**
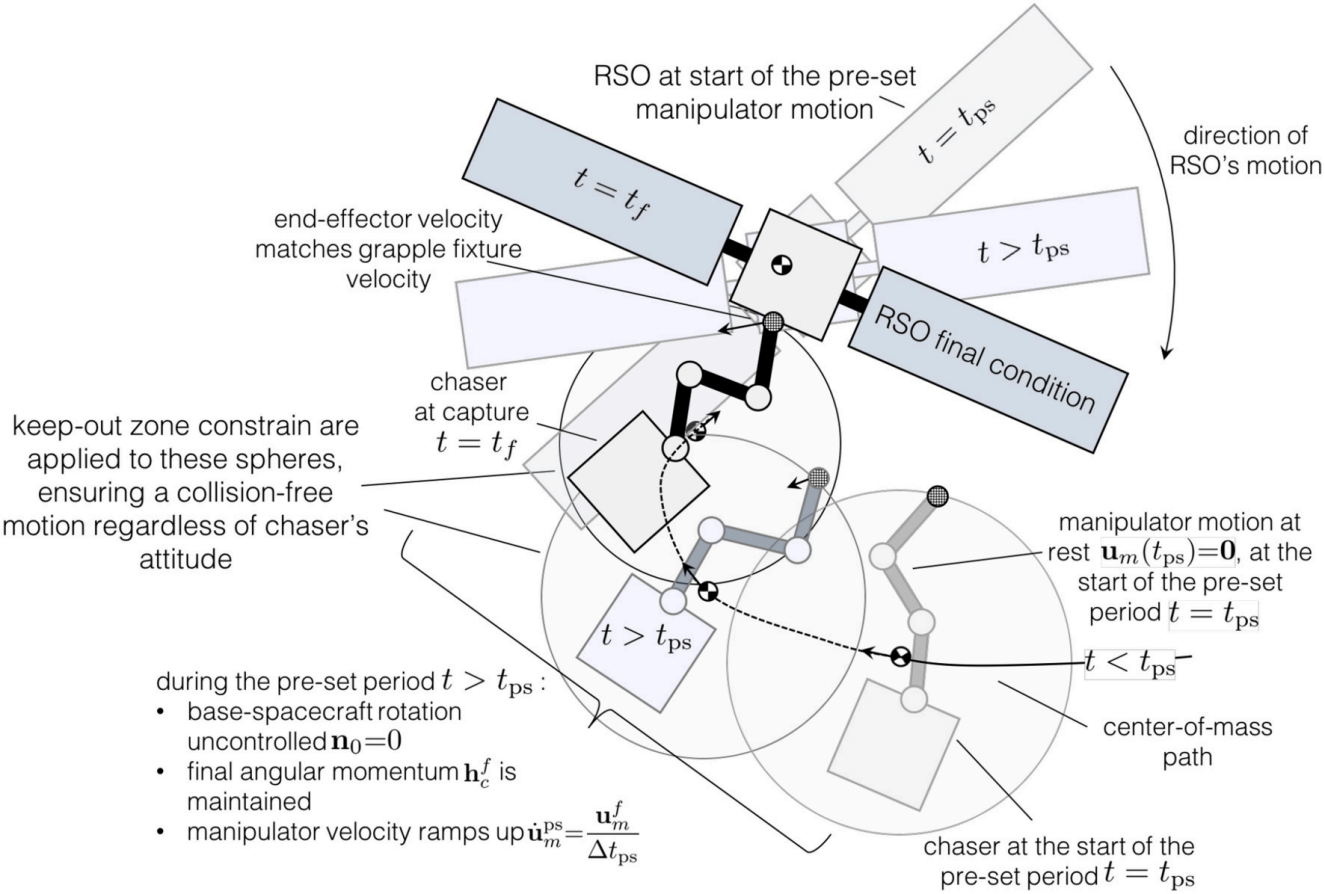
Illustration of the capture constraints and pre-set period motion.

To reduce the control effort of the chaser, the base-spacecraft is operated, during the pre-set manipulator motion period, in a translation-flying/rotation-floating mode, where the base-spacecraft attitude is, for trajectory planning purposes, left uncontrolled. Figure 2 illustrates and summarizes the motion during the pre-set period.

(42) $$\begin{array}{l} {\text{n}}_{0}\left(t\right)=0\text{ for }t\in \left[t_{\text{ps}},t_{f}\right] \end{array}$$

### 3.3. Decelerating and Momenta Transfer

Immediately after the smooth, impact-free capture, the manipulator velocity is decreased by applying, for example, a constant deceleration ${\dot{\text{u}}}_{m}$. During this post-capture deceleration phase, the momenta between the chaser and target RSO are equalized. The momenta transfer is performed via contact forces applied by the chaser's gripper (or by joint forces and torques to transfer momenta within the chaser's multibody system). At capture, the relative linear and angular momenta are stored as internal motion within the newly formed chaser-target multibody system. During the deceleration period, the forces applied to decelerate the manipulator equalize the momenta between the different bodies. As the momenta are gradually equalized, no impact is perceived by either vehicle.

Despite the nominally smooth, impact-free capture, the momenta equalization between the chaser and the target relies on contact forces between the chaser's gripper and the target's grapple fixture. Depending on the mass and inertia of the two vehicles, as well as the tumbling state and momenta transfer rates, these forces could be large, limiting the applicability of this technique or requiring a gripper able to sustain these large forces (Nanjangud et al., 2018). A thorough analysis of the requirements imposed to the chaser's manipulator falls outside the scope of this research.

It is also important to note that in the simultaneous capture and detumbling maneuver the resulting linear momentum is not constrained. As a result, the combined chaser-target system exhibits a residual linear velocity after the maneuver is completed.

(43) $$\begin{array}{l} {\text{p}}_{\text{comb}}\neq 0\;\Rightarrow \;{\dot{\text{r}}}_{\text{comb}}={\dot{\text{r}}}_{r}={\dot{\text{r}}}_{c}\neq 0\;\text{ for }t>t_{f} \end{array}$$

## 4. Guidance of the Simultaneous Capture and Detumble Maneuver as a Collection of Convex Programming Problems

**Figure 3:**
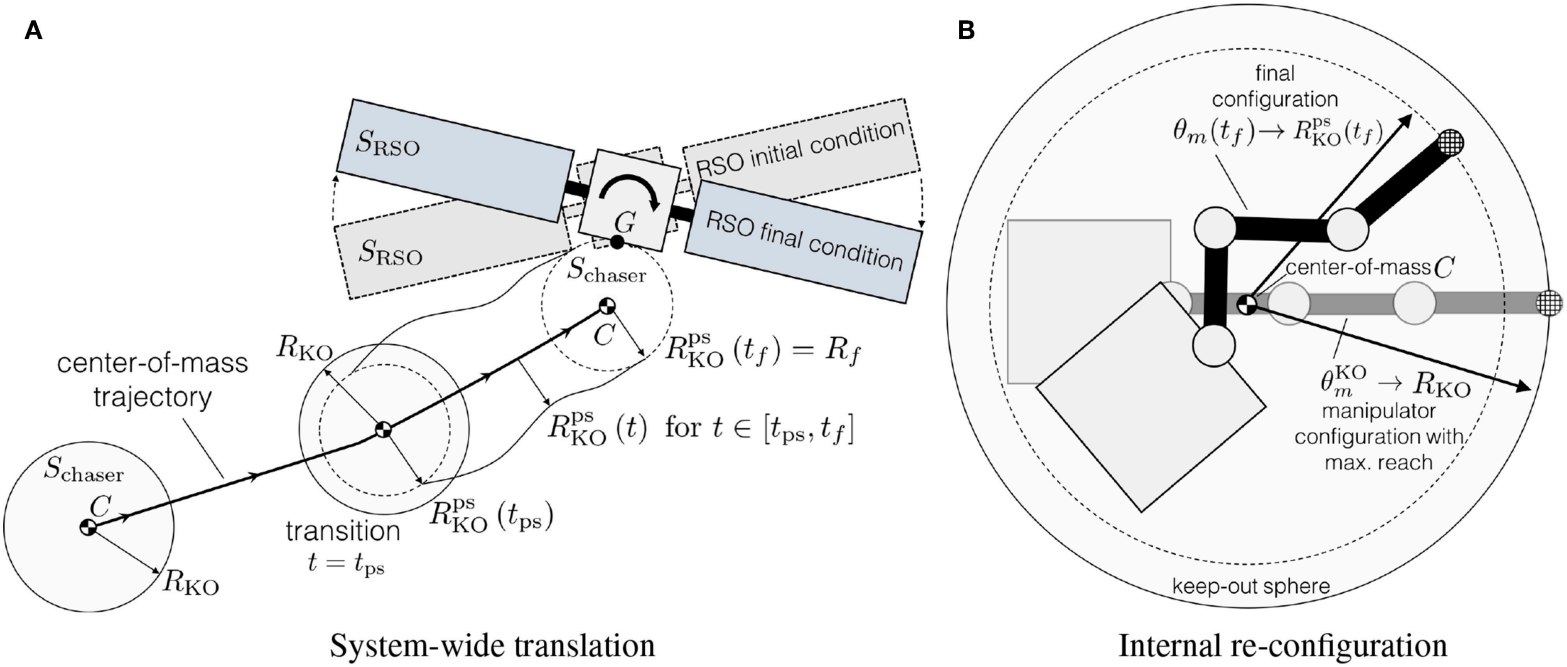
Sub-maneuvers. **(A)** System-wide translation. **(B)** Internal re-con-guration.

For guidance purposes, the chaser's free-flying maneuver is divided into the two concurrent sub-maneuvers shown in Figure 3: a system wide translation and an internal re-configuration. The system-wide translation concerns only the motion of the chaser's center-of-mass, while the internal re-configuration focuses on the manipulator motion and base-spacecraft re-orientation around the chaser's center-of-mass. The complete maneuver is recovered as the combination of the two sub-maneuvers.

The guidance of these two sub-maneuvers is formulated as two consecutive optimization steps, solved via two sequential convex programming procedures. In a sequential convex programming procedure (Virgili-Llop and Romano, 2018), a convex approximation of a non-convex programming problem, formed around the previous iteration solution $\tilde{\left(\cdot \right)}$, is repeatedly solved until the cost, *J*, between two consecutive iterations, ^[*k*−1]^*J*^⋆^ and ^[*k*]^*J*^⋆^, decreases below a certain threshold ϵ:

(44) ‖J[k−1]⋆−J[k]⋆‖1≤ϵ

This guidance approach, first proposed by Virgili-Llop et al. (2017b,c, 2019) for a capture-only maneuver, is here adapted for the simultaneous capture and detumble maneuver.

### 4.1. Step 1: Optimization of the System-Wide Translation

To cast the guidance of the system-wide translation into a convex programming problem, the maneuver time, *t*_*f*_, is fixed and the sub-maneuver transcribed with a direct transcription method (Hull, 1997; Conway, 2012; Sagliano, 2017). A total of *N*_1_ nodes are used, with each individual node denoted by *n*.

The dynamics of the system-wide translation are expressed in a state-space form as,

(45) $$\begin{array}{l} {\text{x}}^{\left[n+1\right]}=\Phi_{\text{r}}^{\left[n\right]}{\text{x}}^{\left[n\right]}+\Psi_{\text{r}}^{\left[n\right]}{\text{f}}_{0}^{\left[n\right]} \end{array}$$

(46) $$\begin{array}{l} {\text{x}}^{\left[n\right]}=\left[\begin{array}{c} {\text{r}}_{c}^{\left[n\right]}\\ {\dot{\text{r}}}_{c}^{\left[n\right]} \end{array}\right] \end{array}$$

with the state transition matrix $\Phi_{\text{r}}^{\left[n\right]}$ and control matrix $\Psi_{\text{r}}^{\left[n\right]}$ defined as follows:

(47) $$\begin{array}{l} \Phi_{\text{r}}^{\left[n\right]}=\left[\begin{array}{cc} {\text{I}}_{3} & \Delta t^{\left[n\right]}{\text{I}}_{3}\\ 0_{3\times 3} & {\text{I}}_{3} \end{array}\right]\;\Psi_{\text{r}}^{\left[n\right]}=\frac{1}{m_{c}}\left[\begin{array}{c} \frac{{\left(\Delta t^{\left[n\right]}\right)}^{2}}{2}{\text{I}}_{3}\\ \Delta t^{\left[n\right]}{\text{I}}_{3} \end{array}\right] \end{array}$$

(48) $$\begin{array}{l} \Delta t^{\left[n\right]}=t^{\left[n+1\right]}-t^{\left[n\right]} \end{array}$$

with ***I***_3_ and **0**_3 × 3_ denoting a 3 × 3 identity and zero matrix, respectively.

The cost to be minimized is formulated as the following quadratic expression, with ${\text{W}}_{1}\in {\mathbb{R}}^{3\times 3}$ denoting the positive semi-definite weight matrix:

(49) $$\begin{array}{l} J_{1}=\overset{N_{1}-1}{\underset{n=1}{\sum }}{{\text{f}}_{0}^{\left[n\right]}}^{T}{\text{W}}_{1}{\text{f}}_{0}^{\left[n\right]}\Delta t^{\left[n\right]} \end{array}$$

In order to satisfy the chaser's control limits, a maximum force constraint is enforced:

(50) $$\begin{array}{l} \left\|{\text{f}}_{0}\right\|\leq f_{0\text{max}} \end{array}$$

Additionally, in order to avoid colliding with the target, a keep-out zone constraint is used. This constraint ensures that the chaser, enclosed by the set *S*_chaser_, and the target RSO, enclosed by the set *S*_RSO_, don't collide. As the chaser's internal configuration is undefined during this first optimization step, *S*_chaser_ is modeled as a sphere, centered around the chaser's center-of-mass *C* and enclosing all admissible internal configurations, **θ**_*m*_, ***q***_0_. As illustrated in Figure 3B, there is a configuration $\theta_{m}^{\text{KO}}$ that defines the maximum radius *R*_KO_. As illustrated in Figure 3A, during the pre-set period of the maneuver, *t* ∈ [*t*_ps_, *t*_*f*_], the manipulator motion is known and a smaller radius, $R_{\text{KO}}^{\text{ps}}\left(t\right)$, is used.

(51) $$\begin{array}{l} S_{\text{chaser}}=\{\begin{array}{l} \left\{r\in {\mathbb{R}}^{3}|\left\|r-r_{c}\right\|\leq R_{\text{KO}}\right\}t\in [0,t_{\text{ps}})\\ \left\{r\in {\mathbb{R}}^{3}|\left\|r-r_{c}\right\|\leq R_{\text{KO}}^{\text{ps}}\left(t\right)\right\}t\in \left[t_{\text{ps}},t_{f}\right] \end{array} \end{array}$$

**Figure 4:**
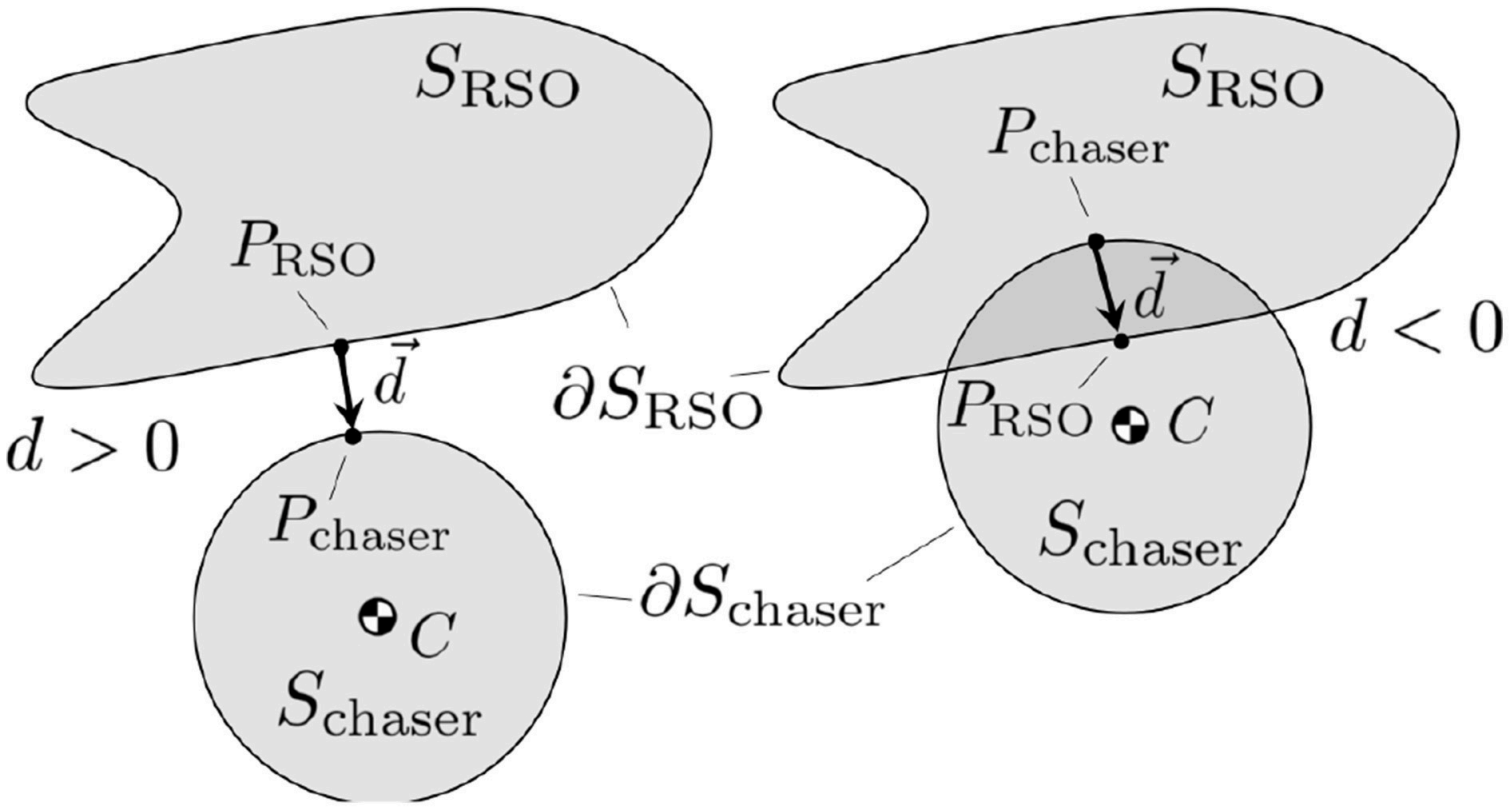
Signed distance *d* and the $\vec{d}$.

Between *S*_chaser_ and *S*_RSO_ a signed distance *d* is defined. If the two sets are not in contact *d* is positive and it is computed as the smallest distance required to bring the two sets in contact. If the two sets intersect *d* is negative and it is computed as the smallest distance required to get the two sets out of contact (Schulman et al., 2014). Then, the $\vec{d}$ vector is defined as the vector between the two closest supporting points *P*_chaser_ ∈ ∂*S*_chaser_ and *P*_RSO_ ∈ ∂*S*_RSO_, as illustrated in Figure 4.

A convex approximation of the keep-out zone constraint, *d* > 0, is found by linearizing the constraint around the previous iteration solution ${\tilde{\text{r}}}_{c}$ (Schulman et al., 2014):

(52) $$\begin{array}{l} \tilde{d}+{\hat{\text{n}}}_{\tilde{\text{d}}}^{T}\left({\text{r}}_{c}-{\tilde{\text{r}}}_{c}\right)\geq 0 \end{array}$$

(53) $$\begin{array}{l} {\hat{\text{n}}}_{\tilde{\text{d}}}=\frac{\tilde{\text{d}}}{\left\|\tilde{\text{d}}\right\|} \end{array}$$

with the quantities with $\tilde{\left(\cdot \right)}$ being computed using the previous iteration's solution ${\tilde{\text{r}}}_{c}$.

In order to complete the system-wide translation sub-maneuver's convex programming problem, the simultaneous capture detumble maneuver center-of-mass position and velocity terminal constraints, Equations (21) and (23), are enforced. **Problem 1. System-wide convex programming problem to be sequentially solved**.

(49, repeated) $$\begin{array}{l} \text{min}:\sum_{n=1}^{N_{1}-1}f_{0}^{{\left[n\right]}^{T}}W_{1}f_{0}^{\left[n\right]}\Delta t^{\left[n\right]} \end{array}$$

(45, repeated) $$\begin{array}{l} \text{s}.\text{t}:\;x^{\left[n+1\right]}=\Phi_{r}^{\left[n\right]}x^{\left[n\right]}+\Psi_{r}^{\left[n\right]}f_{0}^{\left[n\right]}\;n=1\ldots N_{1}-1 \end{array}$$

(50, repeated) $$\begin{array}{l} \left\|f_{0}^{\left[n\right]}\right\|\leq f_{0\text{ max}}\;n=1\ldots N_{1}-1 \end{array}$$

(52, repeated) $$\begin{array}{l} {\tilde{d}}^{\left[n\right]}+{\hat{n}}_{{\;}_{\tilde{d}}}^{[n]T}\left(r_{c}^{\left[n\right]}-{\tilde{r}}_{c}^{\left[n\right]}\right)\geq 0\;n=1\ldots N_{1} \end{array}$$

(21, repeated) $$\begin{array}{l} r_{c}^{\left[n\right]}=r_{c}^{f}\;n=N_{1} \end{array}$$

(23, repeated) $$\begin{array}{l} {\dot{r}}_{c}^{\left[n\right]}={\dot{r}}_{c}^{f}\;n=N_{1} \end{array}$$

Notably, the reference trajectory, ${\tilde{\text{r}}}_{c}$, required to linearize the keep-out zone constraint is not available when the problem is solved for the first time (*k* = 1). A seed to the sequential convex programming procedure is obtained during the first iteration, *k* = 1, by removing the keep-out zone constraint in Equation (52) from the convex programming problem.

The sequential convex programming procedure of the system-wide translation enjoys guaranteed convergence to a locally optimal point—meeting the Karush-Kuhn-Tucker conditions (Boyd and Vandenberghe, 2004)—even if the RSO's shape, *S*_RSO_, is non-convex (Virgili-Llop et al., 2019). To obtain these convergence properties replace, during the second iteration *k* = 2, the RSO shape, *S*_RSO_, by its convex hull. The solution to this problem is an admissible point to the original non-convex problem and, if feasible, subsequent programming problems are bound to remain feasible with a non-strictly decreasing cost (descending algorithm). From the third iteration onwards, *k* ≥ 3, *S*_RSO_ is decomposed into a collection of overlapping convex sets and the linearized keep-out zone constraint is enforced against all these convex sets.

### 4.2. Step 2: Optimization of the Internal Re-Configuration

In order to facilitate the optimization of the internal re-configuration sub-maneuver it is convenient to formulate the multibody dynamics within a non-inertial CCS C, which is assumed to have its origin on the chaser's center-of-mass and its axes parallel to the ones of the inertial CCS I. The equations of motion in the C CCS take the following form:

(54) $$\begin{array}{l} \text{H}{|}_{C}\dot{\text{u}}{|}_{C}+\text{C}{|}_{C}\text{u}{|}_{C}=\tau {|}_{C} \end{array}$$

Note that the inertia matrices and geometric Jacobians in the relative CCS C are equivalent to the ones with respect to the inertial CCS I:

(55) $$\begin{array}{l} \text{H}{|}_{C}=\text{H},\;\text{C}{|}_{C}=\text{C},\;\text{J}{|}_{C}=\text{J} \end{array}$$

As C is non-inertial, a set of generalized inertial forces $\tau_{I}$, derived from the frame's acceleration, appear:

(56) $$\begin{array}{l} \tau {|}_{C}=\tau +\tau_{I} \end{array}$$

These inertial forces, $\tau_{I}$, are the reaction to the center-of-mass forces, ***f***_*c*_ = ***f***_0_, mapped into their equivalent generalized forces. The kineto-static duality is used to obtain this mapping (Siciliano et al., 2009):

(57) $$\begin{array}{l} \tau_{I}=-{\text{J}}_{c}^{T}{\text{f}}_{0} \end{array}$$

(58) $$\begin{array}{l} {\text{J}}_{c}=\frac{\sum_{i=0}^{i=n_{\text{DoF}}}{\text{J}}_{i}m_{i}}{\sum_{i=0}^{i=n_{\text{DoF}}}m_{i}} \end{array}$$

with *m*_*i*_ and ***J***_*i*_ denoting the mass and geometric Jacobian of the *i*th link, respectively.

By definition, the portion of the Jacobian mapping the forces applied to the center-of-mass, ***f***_*c*_, to forces applied to the base-spacecraft, ***f***_0_ is an identity. Therefore, it follows that ${\text{f}}_{0,I}=-{\text{f}}_{0}$. Taking into account this relationship and the identities in Equation (55) the equations of motion in the C CCS can be re-written as:

(59) $$\begin{array}{l} \text{H}\left[\begin{array}{c} {\ddot{\text{r}}}_{0,c}\\ \dot{\bar{\text{u}}} \end{array}\right]+\text{C}\left[\begin{array}{c} {\dot{\text{r}}}_{0,c}\\ \bar{\text{u}} \end{array}\right]=\left[\begin{array}{c} 0_{3\times 1}\\ \bar{\tau }-{\bar{\text{J}}}_{c}^{T}{\text{f}}_{0} \end{array}\right] \end{array}$$

(60) $$\begin{array}{l} \dot{\bar{\text{u}}}=\left[\begin{array}{c} {\dot{\omega }}_{0}\\ {\dot{\text{u}}}_{m} \end{array}\right]\;\bar{\text{u}}=\left[\begin{array}{c} \omega_{0}\\ {\text{u}}_{m} \end{array}\right]\;\bar{\tau }=\left[\begin{array}{c} {\text{n}}_{0}\\ \tau_{m} \end{array}\right] \end{array}$$

with $\bar{\tau }\in {\mathbb{R}}^{3+n_{\text{DoF}}}$ denoting the control variables and ${\bar{\text{J}}}_{c}^{T}$ the sub-Jacobian, corresponding to the internal re-configuration sub-maneuver control variable $\bar{\tau }$.

During the pre-set period of the maneuver, the chaser is operated in a translation-flying/rotation-floating control mode, with the base-spacecraft attitude left uncontrolled (see Equation 42). The manipulator motion during the pre-set period is known and the center-of-mass trajectory for the entire maneuver has been obtained during the optimization of the

system wide translation sub-maneuver. Therefore, the attitude motion, **ω**_0_, during the pre-set period can be obtained with Equation (59) and integrated backwards to obtain ***q***_0_ before initiating the internal re-configuration optimization. As the base-spacecraft attitude, **ω**_0_, ***q***_0_, and manipulator motion, ***u***_*m*_, **θ**_*m*_, are known during the pre-set period, the internal re-configuration optimization step only needs to extend until the start of the pre-set period *t* ≤ *t*_ps_. The pre-set period is excluded from the internal re-configuration optimization. Note that during the first optimization step the keep-out zone constraint is applied to a sphere enclosing all possible chaser attitudes. Therefore, even if the attitude is not controlled during the pre-set period, the obstacle avoidance properties, as shown in Figure 2, are preserved.

In this optimization step, a sequential convex programming procedure is used to overcome the nonlinear kinematics and dynamics of the multibody system. A convex approximation of the problem is obtained by linearizing the kinematics and dynamics around the previous iteration solution, denoted by $\tilde{\bar{\text{u}}},{\tilde{\theta }}_{m},{\tilde{\text{q}}}_{0}$. To ensure that the solution remains within the region where the linearization is valid, trust regions around **θ**_*m*_ and **ω**_0_ are enforced:

(61) $$\begin{array}{l} {\left\|\theta_{m}^{\left[n\right]}-{\tilde{\theta }}_{m}^{\left[n\right]}\right\|}_{1}\leq \rho_{\theta_{m}} \end{array}$$

(62) $$\begin{array}{l} {\left\|\omega_{0}^{\left[n\right]}-{\tilde{\omega }}_{0}^{\left[n\right]}\right\|}_{1}\leq \rho_{\omega_{0}} \end{array}$$

with ρ_**θ**_*m*__ and ρ_**ω**_0__ denoting the user-tunable radius of the trust region.

A direct transcription method with *N*_2_ nodes is used to transcribe the sub-maneuver, with the generalized accelerations ${\dot{\bar{\text{u}}}}^{\left[n\right]}$ serving as the optimization variables. A trapezoidal integration scheme is used to obtain the quadratic cost for this sub-maneuver with ${\text{W}}_{2}\in {\mathbb{R}}^{\left(n_{\text{DoF}}+3\right)\times \left(n_{\text{DoF}}+3\right)}$ denoting the positive semi-definite weight matrix.

(63) $$\begin{array}{l} J_{2}=\overset{N_{2}-1}{\underset{n=1}{\sum }}\frac{{{\bar{\tau }}^{\left[n+1\right]}}^{T}{\text{W}}_{2}{\bar{\tau }}^{\left[n+1\right]}-{{\bar{\tau }}^{\left[n\right]}}^{T}{\text{W}}_{2}{\bar{\tau }}^{\left[n\right]}}{2}\Delta t^{\left[n\right]} \end{array}$$

The velocities, $\bar{\text{u}}$, and manipulator joint displacements, **θ**_*m*_, can be propagated using state transition matrices.

(64) $$\begin{array}{l} {\bar{\text{u}}}^{\left[n+1\right]}=\Phi_{\text{u}}{\bar{\text{u}}}^{\left[n\right]}+\Theta_{\text{u}}^{\left[n\right]}{\dot{\bar{\text{u}}}}^{\left[n\right]} \end{array}$$

(65) $$\begin{array}{l} \Phi_{\text{u}}={\text{I}}_{3+n_{\text{DoF}}} \end{array}$$

(66) $$\begin{array}{l} \Theta_{\text{u}}^{\left[n\right]}=\Delta t^{\left[n\right]}{\text{I}}_{3+n_{\text{DoF}}} \end{array}$$

(67) $$\begin{array}{l} \theta_{m}^{\left[n+1\right]}=\Phi_{\theta }\theta_{m}^{\left[n\right]}+\Theta_{\theta }^{\left[n\right]}\left[\begin{array}{c} {\text{u}}_{m}^{\left[n\right]}\\ {\dot{\text{u}}}_{m}^{\left[n\right]} \end{array}\right] \end{array}$$

(68) $$\begin{array}{l} \Phi_{\theta }={\text{I}}_{n_{\text{DoF}}} \end{array}$$

(69) $$\begin{array}{l} \Theta_{\theta }^{\left[n\right]}=\left[\begin{array}{cc} \Delta t^{\left[n\right]}{\text{I}}_{n_{\text{DoF}}} & \frac{{\left(\Delta t^{\left[n\right]}\right)}^{2}}{2}{\text{I}}_{n_{\text{DoF}}} \end{array}\right] \end{array}$$

The linear approximation of the quaternion differential kinematics is obtained as follows:

(70) $$\begin{array}{l} \omega_{0}^{\left[n+1/2\right]}=\frac{\omega_{0}^{\left[n+1\right]}+\omega_{0}^{\left[n\right]}}{2} \end{array}$$

(71) $$\begin{array}{l} {\hat{\omega }}_{0}^{\left[n+1/2\right]}=\frac{\omega_{0}^{\left[n+1/2\right]}}{\|\omega_{0}^{\left[n+1/2\right]}\|} \end{array}$$

(72) $$\begin{array}{l} \alpha_{\omega_{0}}^{\left[n+1/2\right]}=\left\|\omega_{0}^{\left[n+1/2\right]}\right\|\frac{\Delta t^{\left[n\right]}}{2} \end{array}$$

(73) $$\begin{array}{l} f_{\text{q}}^{\left[n\right]}\left({\text{q}}_{0}^{\left[n\right]},\omega_{0}^{\left[n+1/2\right]}\right)=\left[\begin{array}{c} {\hat{\omega }}_{0}^{\left[n+1/2\right]}\sin\alpha_{\omega_{0}}^{\left[n+1/2\right]}\\ \cos\alpha_{\omega_{0}}^{\left[n+1/2\right]} \end{array}\right]⊗{\text{q}}_{0}^{\left[n\right]} \end{array}$$

(74) $$\begin{array}{l} {\text{q}}_{0}^{\left[n+1\right]}\approx {\tilde{\text{q}}}_{0}^{\left[n+1\right]}+\nabla f_{\text{q}}\left[\begin{array}{c} {\text{q}}_{0}^{\left[n\right]}-{\tilde{\text{q}}}_{0}^{\left[n\right]}\\ \omega_{0}^{\left[n+1/2\right]}-{\tilde{\omega }}_{0}^{\left[n+1/2\right]} \end{array}\right] \end{array}$$

The following partial linearization of the multibody dynamics is used:

(75) $$\begin{array}{l} \bar{\tau }\approx -{\tilde{\bar{\tau }}}_{I}+\tilde{\bar{\text{H}}}\left[\begin{array}{c} {\ddot{\text{r}}}_{0}{|}_{C}-{\tilde{\ddot{\text{r}}}}_{0}{|}_{C}\\ \dot{\bar{\text{u}}}-\tilde{\dot{\bar{\text{u}}}} \end{array}\right]+\tilde{\bar{\text{C}}}\left[\begin{array}{c} {\dot{\text{r}}}_{0}{|}_{C}-{\tilde{\dot{\text{r}}}}_{0}{|}_{C}\\ \bar{\text{u}}-\tilde{\bar{\text{u}}} \end{array}\right] \end{array}$$

Limits on the joint displacements, joint forces, and base-spacecraft torques are enforced:

(76) $$\begin{array}{l} \theta_{m\text{min}}\leq \theta_{m}\leq \theta_{m\text{max}} \end{array}$$

(77) $$\begin{array}{l} \tau_{m\text{min}}\leq \tau_{m}\leq \tau_{m\text{max}} \end{array}$$

(78) $$\begin{array}{l} \left\|{\text{n}}_{0}\right\|\leq n_{0\text{max}} \end{array}$$

and the entry conditions to the pre-set period are used as the terminal constraints.

(79) $$\begin{array}{l} \theta_{m}^{\left[n\right]}=\theta_{m}^{\text{ps}}\left(t_{\text{ps}}\right)\;n=N_{2} \end{array}$$

(80) $$\begin{array}{l} {\text{u}}_{m}^{\left[n\right]}={\text{u}}_{m}^{\text{ps}}\left(t_{\text{ps}}\right)\;n=N_{2} \end{array}$$

(81) $$\begin{array}{l} {\text{q}}_{0}^{\left[n\right]}={\text{q}}_{0}^{\text{ps}}\left(t_{\text{ps}}\right)\;n=N_{2} \end{array}$$

(82) $$\begin{array}{l} \omega_{0}^{\left[n\right]}=\omega_{0}^{\text{ps}}\left(t_{\text{ps}}\right)\;n=N_{2} \end{array}$$

**Problem 2. Internal re-configuration convex programming problem to be sequentially solved**.

(63, repeated) $$\begin{array}{l} \text{min}:\sum_{n=1}^{N_{2}-1}\frac{{\bar{\tau }}^{[n+1]T}W_{2}{\bar{\tau }}^{\left[n+1\right]}-{\bar{\tau }}^{[n]T}W_{2}{\bar{\tau }}^{\left[n\right]}}{2}\Delta t^{\left[n\right]} \end{array}$$

(75, repeated) $$\begin{array}{l} \text{s}.\text{t}.:{\bar{\tau }}^{\left[n\right]}=-{\tilde{\bar{\tau }}}_{Itexkk}^{\left[n\right]}+{\tilde{\bar{H}}}^{\left[n\right]}\left[\begin{array}{l} {\ddot{r}}_{0,c}^{\left[n\right]}-{\tilde{\ddot{r}}}_{0,c}^{\left[n\right]}\\ {\dot{\bar{u}}}^{\left[n\right]}-{\tilde{\dot{\bar{u}}}}^{\left[n\right]} \end{array}\right]+{\tilde{\bar{C}}}^{\left[n\right]}\left[\begin{array}{l} {\dot{r}}_{0,c}^{\left[n\right]}-{\tilde{\dot{r}}}_{0,c}^{\left[n\right]}\\ {\bar{u}}^{\left[n\right]}-{\tilde{\bar{u}}}^{\left[n\right]} \end{array}\right]\;n=1\ldots N_{2} \end{array}$$

(74, repeated) $$\begin{array}{l} q_{0}^{\left[n+1\right]}={\tilde{q}}_{0}^{\left[n+1\right]}+\nabla f_{q}\left[\begin{array}{c} q_{0}^{\left[n\right]}-{\tilde{q}}_{0}^{\left[n\right]}\\ \omega_{0}^{\left[n+1/2\right]}-{\tilde{\omega }}_{0}^{\left[n+1/2\right]} \end{array}\right]\;n=1\ldots N_{2}-1 \end{array}$$

(64, repeated) $$\begin{array}{l} {\bar{u}}^{\left[n+1\right]}=\Phi_{u}{\bar{u}}^{\left[n\right]}+\Theta_{u}^{\left[n\right]}{\dot{\bar{u}}}^{\left[n\right]}\;n=1\ldots N_{2}-1 \end{array}$$

(67, repeated) $$\begin{array}{l} \theta_{m}^{\left[n+1\right]}=\Phi_{\theta }\theta_{m}^{\left[n\right]}+\Theta_{\theta }^{\left[n\right]}\left[\begin{array}{c} \omega_{m}^{\left[n\right]}\\ {\dot{\omega }}_{m}^{\left[n\right]} \end{array}\right]\;n=1\ldots N_{2}-1 \end{array}$$

(76, repeated) $$\begin{array}{l} \theta_{m\text{min}}\leq \theta_{m}^{\left[n\right]}\leq \theta_{m\text{ max}}\;n=1\ldots N_{2} \end{array}$$

(77, repeated) $$\begin{array}{l} \tau_{m\text{min}}\leq \tau_{m}^{\left[n\right]}\leq \tau_{m\text{max}}\;n=1\ldots N_{2} \end{array}$$

(78, repeated) $$\begin{array}{l} \left\|n_{0}^{\left[n\right]}\right\|\leq n_{0\text{ max}}\;n=1\ldots N_{2}-1 \end{array}$$

(79, repeated) $$\begin{array}{l} \theta_{m}^{\left[n\right]}=\theta_{m}^{\text{ps}}\left(t_{\text{ps}}\right)\;n=N_{2} \end{array}$$

(80, repeated) $$\begin{array}{l} u_{m}^{\left[n\right]}=u_{m}^{\text{ps}}\left(t_{\text{ps}}\right)\;n=N_{2} \end{array}$$

(81, repeated) $$\begin{array}{l} q_{0}^{\left[n\right]}=q_{0}^{\text{ps}}\left(t_{\text{ps}}\right)\;n=N_{2} \end{array}$$

(82, repeated) $$\begin{array}{l} \omega_{0}^{\left[n\right]}=\omega_{0}^{\text{ps}}\left(t_{\text{ps}}\right)\;n=N_{2} \end{array}$$

(61, repeated) $$\begin{array}{l} {\left\|\theta_{m}^{\left[n\right]}-{\tilde{\theta }}_{m}^{\left[n\right]}\right\|}_{1}\leq \rho_{\theta_{m}}\;n=1\ldots N_{2} \end{array}$$

(62, repeated) $$\begin{array}{l} {\left\|\omega_{0}^{\left[n\right]}-{\tilde{\omega }}_{0}^{\left[n\right]}\right\|}_{1}\leq \rho_{\omega_{0}}\;n=1\ldots N_{2} \end{array}$$

The convex approximation of the problem relies on a linearization around a set trajectory $\tilde{\bar{\text{u}}},{\tilde{\theta }}_{m},{\tilde{\text{q}}}_{0}$. The first time the problem is solved there is no previous solution to rely on and an initial guess is required. An initial guess for the base-spacecraft's attitude and manipulator's motion can be obtained assuming that the base-spacecraft performs an eigenaxis maneuver and the manipulator follows a linear trajectory in joint space (i.e., minimum deflection from initial to final configuration).

### 4.3. Selecting the Final Base-Spacecraft Attitude ${\text{q}}_{0}^{f}$

**Figure 5:**
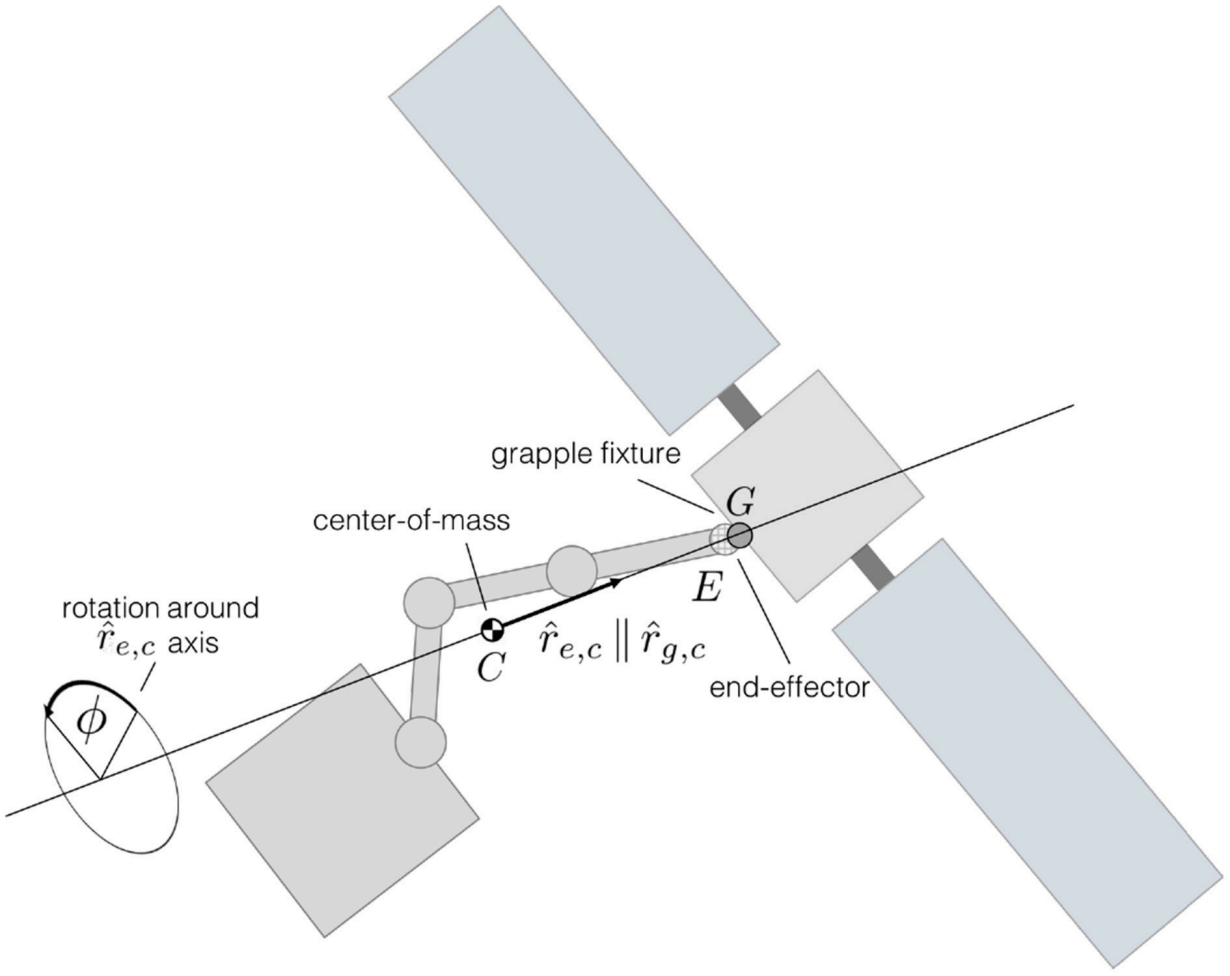
The angular displacement about the axis connecting the center-of-mass and the end-effector/grapple fixture is undetermined.

The final position of the chaser's center-of-mass is set by the pre-set final manipulator configuration $\theta_{m}^{f}$. This condition, partially specifies the attitude of the chaser, yet leaving undetermined an angular displacement, ϕ, about the ${\hat{\text{r}}}_{e,c}$ axis, as shown in Figure 5.

During preliminary simulations it became apparent that small manipulator velocities increase the probability of obtaining feasible maneuvers, indicating that $\left\|{\text{u}}_{m}^{f}\right\|$ is a proxy for the maneuver's complexity. The manipulator velocities corresponding to several ϕ displacements can be computed, and the angular displacement, ϕ, that produces the minimum $\left\|{\text{u}}_{m}^{f}\right\|$ can be used to fully define the base-spacecraft capture attitude, and by extension, the manipulator and base-spacecraft attitude motion during the pre-set period.

### 4.4. Selecting the Initial and Final Time

In order to cast both sub-maneuver optimization problems as convex programming problems, they are converted to fixed-final-time problems. As discussed by Virgili-Llop et al. (2019), and later shown in section 5, longer final times, *t*_*f*_, reduce, in broad terms, the cost of the maneuver. The user is then prompted to select the longest possible maneuver duration, only limited by the available computing power, or by other factors as communication, or illumination windows. Once the maneuver time is fixed, the following question arises: When does the maneuver start? The window when the maneuver can occur, limited by illumination, communication, or other factors, may be longer than the actual maneuver duration. Instead of starting the maneuver right at the beginning of the window, it may be advantageous to wait for more favorable capture conditions. As the manipulator velocity is a proxy for the capture complexity, a capture condition inducing low manipulator velocities is, in general, desired. The chaser can analyze the capture conditions at different times during the capture window, and start the maneuver when the smallest manipulator velocity at capture is anticipated. The numerical simulations shown in section 5 indicate that this strategy helps to increase the ability of the guidance algorithm to find feasible solutions.

## 5. Numerical Simulations

**Figure 6:**
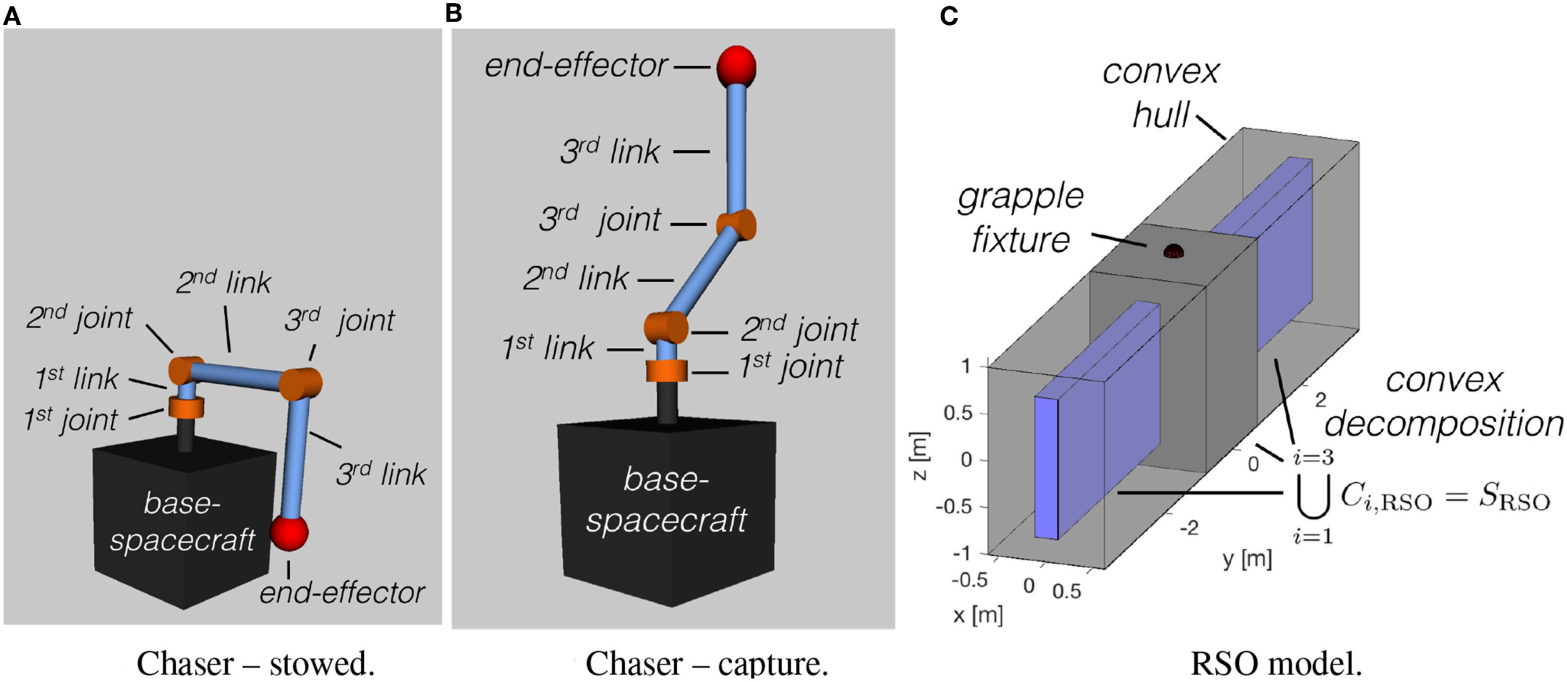
Chaser and RSO models used in the numerical simulations. **(A)** Chaser stowed. **(B)** Chaser capture. **(C)** RSO model.

In order to illustrate the proposed simultaneous capture and detumble maneuver, a numerical simulation case study is provided. The chaser and target RSO used are shown in Figure 6. The parameters used in the simulations are provided in Table 1. For added insight, a Monte Carlo analysis was conducted for 11 maneuver times (*t*_*f*_) and 11 initial RSO angular velocity magnitudes (∥**ω**_*r*_(*t* = 0)∥). For each pair of *t*_*f*_ and ∥**ω**_*r*_(*t* = 0)∥, 100 simulations with randomized RSO initial orientations (as proposed by Shoemake, 1995) and angular velocity directions have been conducted. For each case two capture maneuvers are simulated, one starting the maneuver right away with the initial random orientation and velocity, while the other allowing the chaser to wait for more favorable capture conditions. A total of 11 × 11 × 100 × 2 = 24, 200 maneuvers have been simulated, allowing to assess the robustness of the proposed guidance. The post-capture manipulator deceleration has not been simulated and it is assumed to be feasible for all cases.

**Table 1:** Numerical simulation parameters.

**Parameter**	**Value**
**Chaser**	
Initial distance from RSO	10 m
Initial velocity	0 m s^-1 & 0 deg/s
Initial orientation	Pointing toward the target RSO
Mass of the base	105 kg
Inertia of the base	*J*_*xx, yy, zz*_ = 9.3 kg m^2
Mass of the links	*m*_1_ = 5, *m*_2_ = 10, and *m*_3_ = 10 kg
Length of the links	*l*_1_ = 0.2, *l*_2_ = 0.75, and *l*_3_ = 0.75 m
Initial configuration **θ**_*m*_(*t*_0_)	θ_1_ = π, $\theta_{2}=\frac{\pi }{2}$, and $\theta_{3}=\frac{\pi }{2}$ (Figure 6A)
Grasping configuration **θ**_*m*_(*t*_*f*_)	θ_1_ = −π, $\theta_{2}=\frac{\pi }{4}$, and $\theta_{3}=-\frac{\pi }{4}$ (Figure 6B)
Grasping pose reach	$R_{\text{KO}}^{\text{ps}}\left(t_{f}\right)=1.90$ m
Max. keep-out sphere radius	*R*_KO_ = 2.04 m
Max. force	*F*_0 max_ = 6.25 N
Max. base torque	τ_0 max_ = 1 N m
Max. joint torque	τ_*m*max_ = 1 N m
Max. joint deflections	θ_1_ = ±π, $\theta_{2}=\pm \frac{\pi }{2}$, and θ_3_ = ±1.75
**RSO**	
Initial attitude	Random
Initial angular velocity	0-10 deg/s with random initial direction
Mass	130 kg
Inertia	*J*_*xx*_ = 98.54, *J*_*yy*_ = 54.84, and *J*_*zz*_ = 72.55 kg m^2
**Optimization**	
Number of nodes	*N*_1,2_ = 101
Max pre-set period max(*t*_*f*_−*t*_ps_)	10 s
First step convergence criteria	ϵ_1_ = 0.01% of J1⋆[k−1]
Second step convergence criteria	ϵ_2_ = 2% of J1⋆[k−2]
Trust regions	ρ_**θ**_*m*__ = 0.5°, ρ_**ω**_0__ ≥ 0.5 deg/s
**Monte Carlo**	
Maneuver time *t*_*f*_	80,82,84,86,88,90,92,94,96,98, 100 s
RSO's initial angular velocity magnitude ∥**ω**_*r*_(*t* = 0)∥	0,1,2,3,4,5,6,7,8,9,10 deg/s
Number of samples per *t*_*f*_, ∥**ω**_*r*_(*t* = 0)∥ combination	100

### 5.1. Numerical Simulation Results

**Figure 7:**
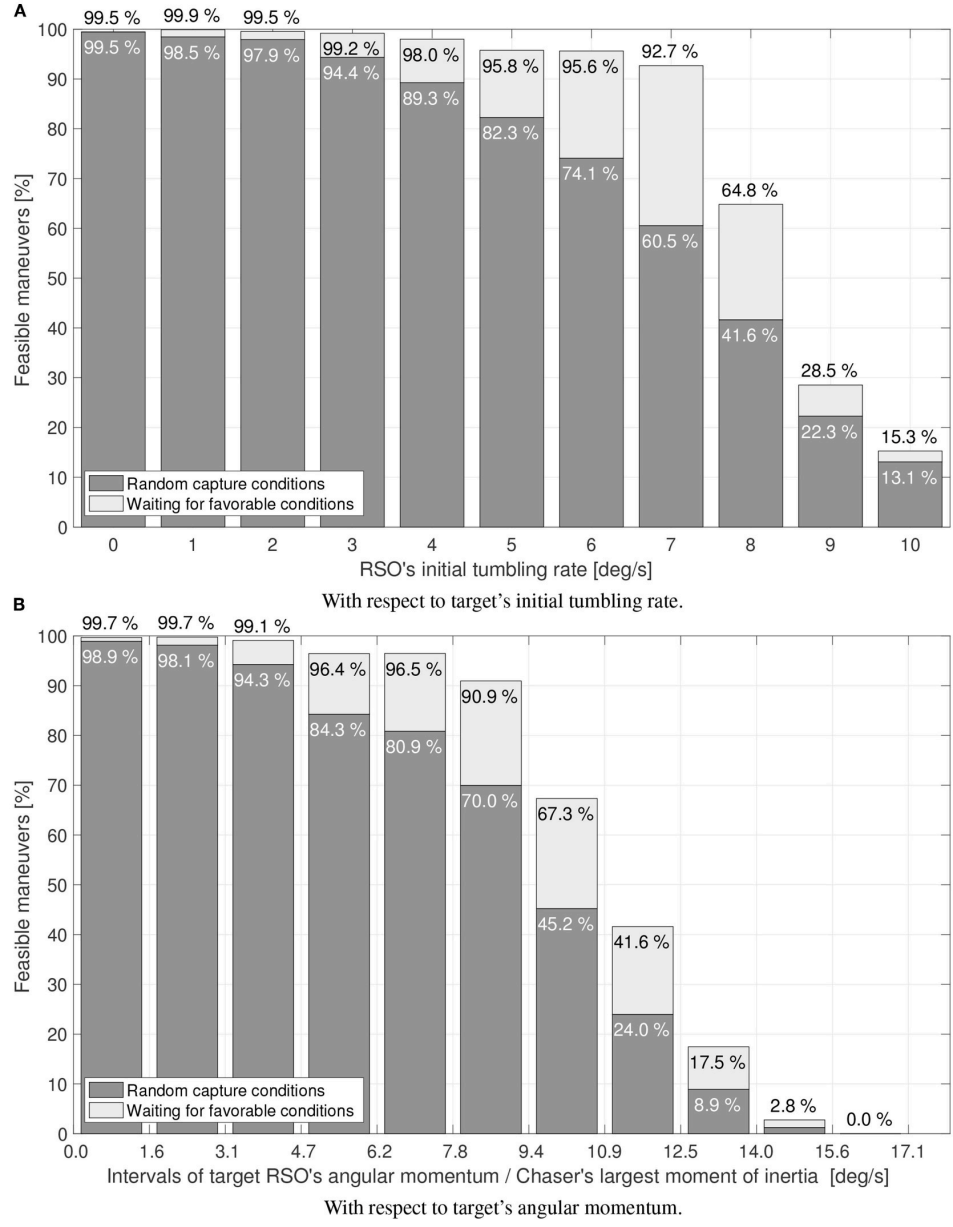
Simulation results: percentage of feasible maneuvers. **(A)** With respect to target's initial tumbling rate. **(B)** With respect to target's angular momentum.

Figure 7 shows the percentage of feasible simultaneous maneuvers found by the proposed guidance algorithm. Figure 7A shows the feasibility with respect to the target RSO initial tumbling rate, while Figure 7B shows the feasibility with respect to the target's angular momentum, normalized by the chaser's largest moment of inertia. The results suggest that the simultaneous capture and detumble maneuver gets increasingly difficult as the RSO's tumbling rate increases.

The strategy of initiating the maneuver right away, or waiting for more favorable conditions within the capture window is compared in Figure 7. The capture window has been assumed to last for one tumbling period (*P* = 2π/‖**ω**_*r*_‖), upper bounded at 5 min. From the results shown in Figure 7, it can be concluded that waiting for more favorable capture conditions significantly increases the guidance ability to find feasible maneuvers.

With the particular combination of chaser and target simulated here, the proposed guidance approach is able to find feasible simultaneous capture and detumble maneuvers for moderate tumbling rates (<7 deg/s) and a wide range of initial RSO states. These feasibility statistics contrast with the remarkable 99.95% success rate obtained by capture-only maneuvers using the matching velocity terminal constraint, which were shown to be insensitive to the RSO's tumbling rate—at least up to 10 deg/s (Virgili-Llop et al., 2019). The simultaneous capture and detumble terminal constraints significantly differ from the matching velocity ones. The terminal constraint enforced on a simultaneous capture and detumble maneuver, produces a capture under relative velocity, only offering an instantaneous capture window. The relative velocity and manipulator velocity at capture (see Equations 23 and 32) increase as the target's angular momentum to be neutralized increases, suggesting that the maneuver becomes more challenging for large target inertias or high tumbling rates. This seems to indicate that the feasibility of the maneuver decreases with increasing RSO's rates and inertia. The broader question of maneuver feasibility, or reachability, is in general very difficult to answer and constitutes its own research field (Zagaris and Romano, 2018).

**Figure 8:**
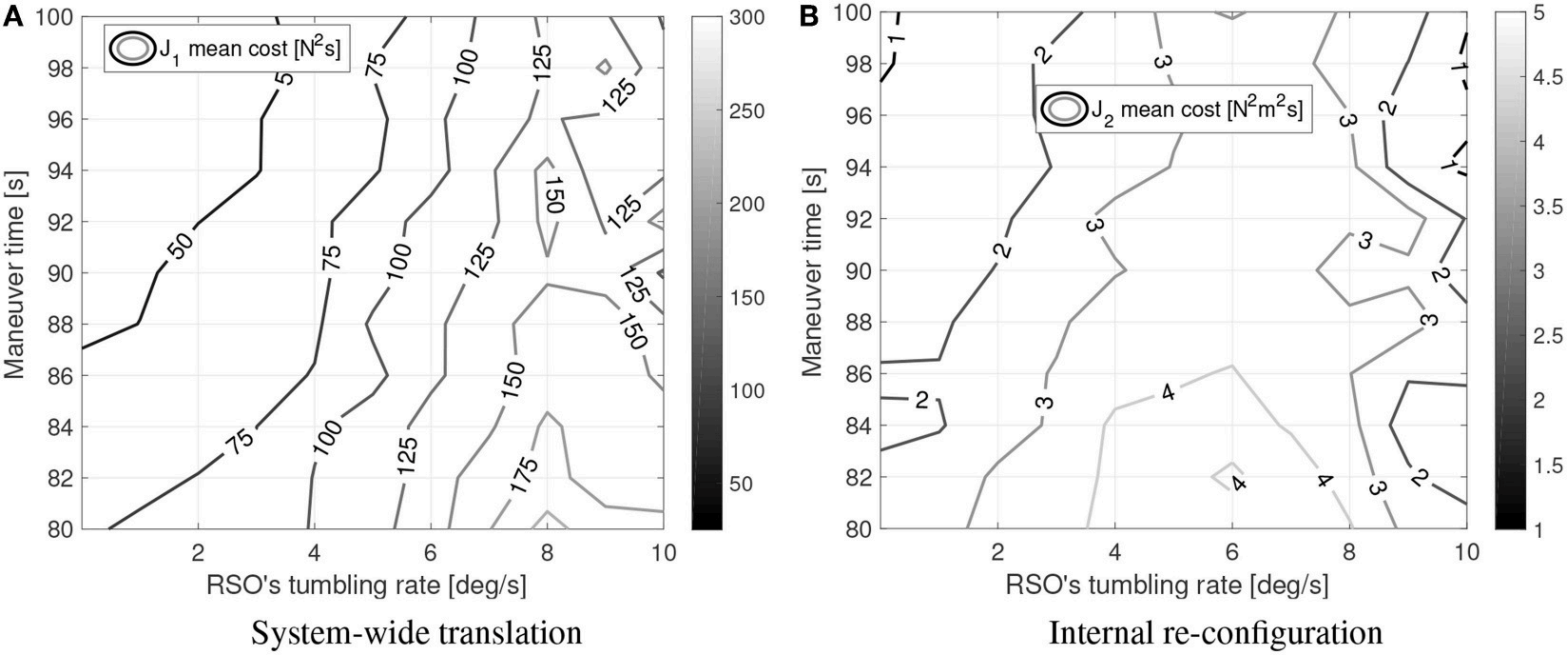
Simulation results: sub-maneuvers mean cost. **(A)** System-wide translation. **(B)** Internal re-con-guration.

**Figure 9:**
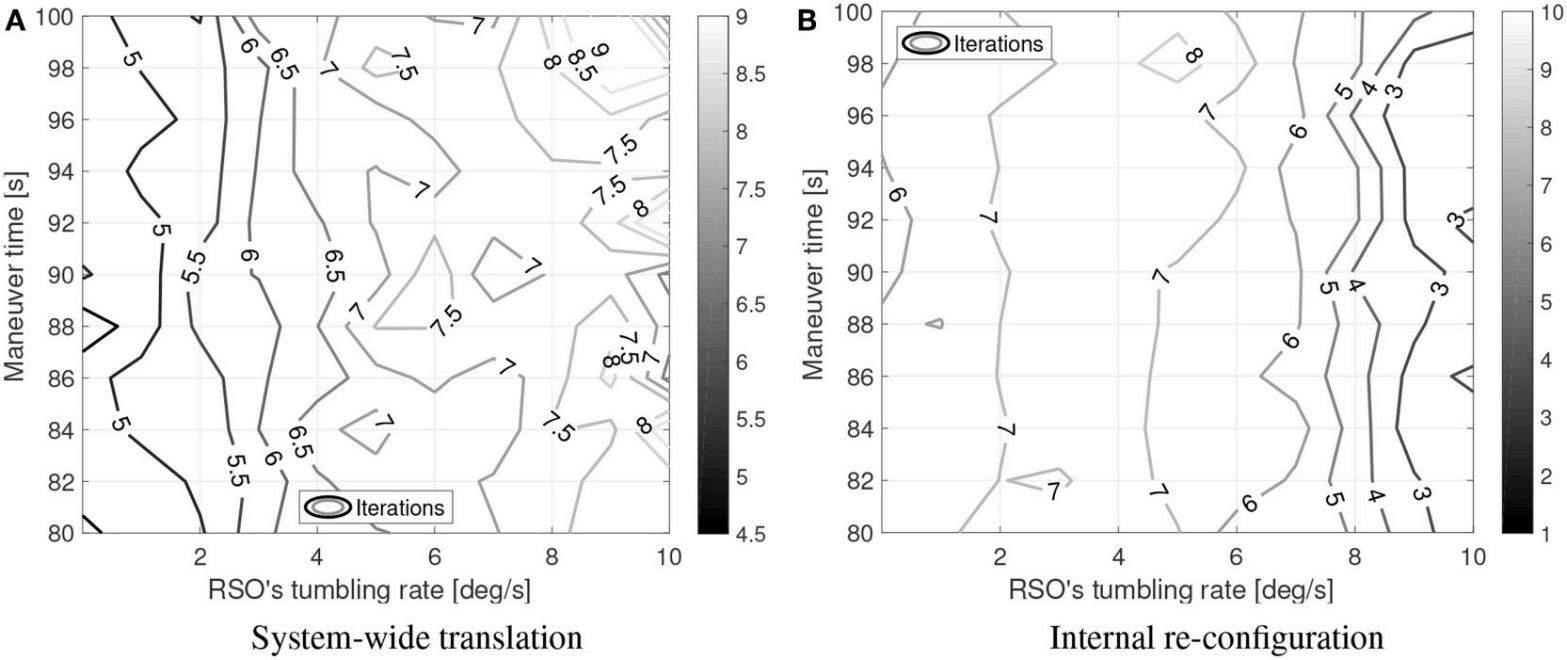
Simulation results: mean iterations to converge. **(A)** System-wide translation. **(B)** Internal re-con-guration.

**Figure 10:**
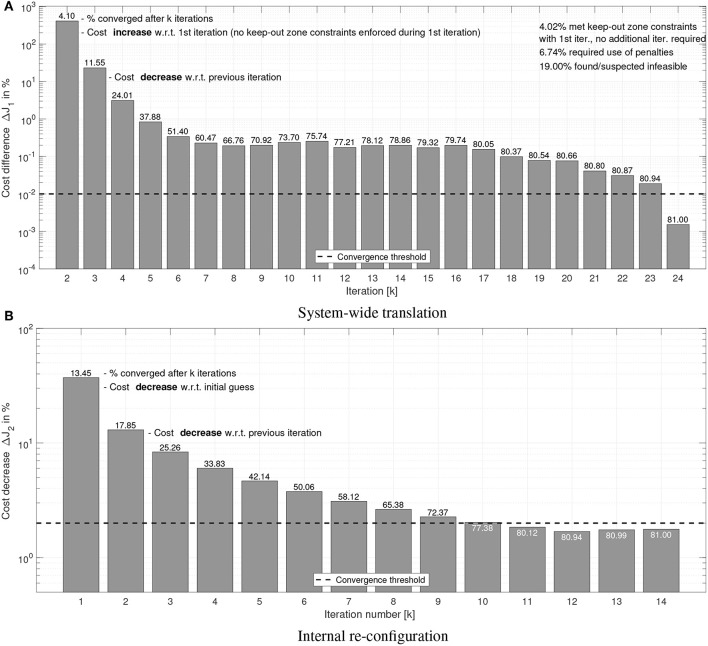
Simulation results: convergence rate. Dashed line shows the convergence threshold. **(A)** System-wide translation. **(B)** Internal re-con-guration.

For the converged cases, Figures 8, 9 show the mean sub-maneuver cost and mean number of iterations required by each sequential convex programming procedure to converge. As expected, the system-wide sub-maneuver cost tends to increase as the tumbling rate increases or the maneuver time decreases. The internal re-configuration sub-maneuver cost exhibits a maximum cost at intermediate tumbling rates while also appearing to decrease for increasing maneuver durations. Figures 10 shows the convergence rate for both optimization steps. From the convergence rate figures it appears that limiting number of iterations of each optimization step to, for example nine, upper-bounds the guidance computation time, without incurring in an excessive loss of optimality (cost decreases after nine iterations are small on both optimization steps).

Figures 8,9 can be compared to their counterparts in Virgili-Llop et al. (2019), where a capture-only maneuver was studied, showing that adding the detumble terminal constraints makes the maneuver more expensive and computationally complex.

A video showing the two sequential convex programming procedures used on both optimization steps is attached as Supplementary Material.

### 5.2. Verification of Assumption A.2

A brief analysis to confirm the validity of the assumption that the environmental and relative orbital dynamics disturbances are small and can be safely neglected is now presented. The order of magnitude of the relative orbital dynamics can be estimated, using the Clohessy-Wiltshire equations of relative motion (Fehse, 2003), by ${\ddot{r}}_{CW}\approx 3n_{o}^{2}r+2n_{o}ṙ$ (with *n*_*o*_ denoting the orbit's mean motion). The results obtained with the numerical simulations, with *r* ≤ 10 m and ṙ ≤ 0.39 m s-1 (on a 10 deg/s tumbling rate case), imply that, at low orbital altitudes (500 km circular orbit), ${\ddot{r}}_{CW}$ is 1.9% of the chaser's maximum acceleration, *F*_0 max_/*m*_*c*_. At geostationary orbital altitudes, ${\ddot{r}}_{CW}$ drops to 0.12% of *F*_0 max_/*m*_*c*_. To bound the effects of differential aerodynamic drag let's assume that the target is operating at low altitudes (500 km circular orbit), orbiting through a residual atmosphere with a density of 10^−11^ kg m^−3^. Additionally, let's assume that the target's cross section area is *A* = 12 m^2^ and with a drag coefficient *C*_*d*_ = 2.2. With these conditions, the target would be subjected to 0.008 N of drag. If the chaser did not experience any drag—worst-case scenario—the differential aerodynamic drag would represent 0.12% of *F*_0 max_. For the solar radiation pressure a similar analysis can be conducted, showing that a worst-case solar radiation pressure differential would amount to 0.002% of *F*_0 max_.

## 6. Experiments on a Planar Air Bearing Table

**Figure 11:**
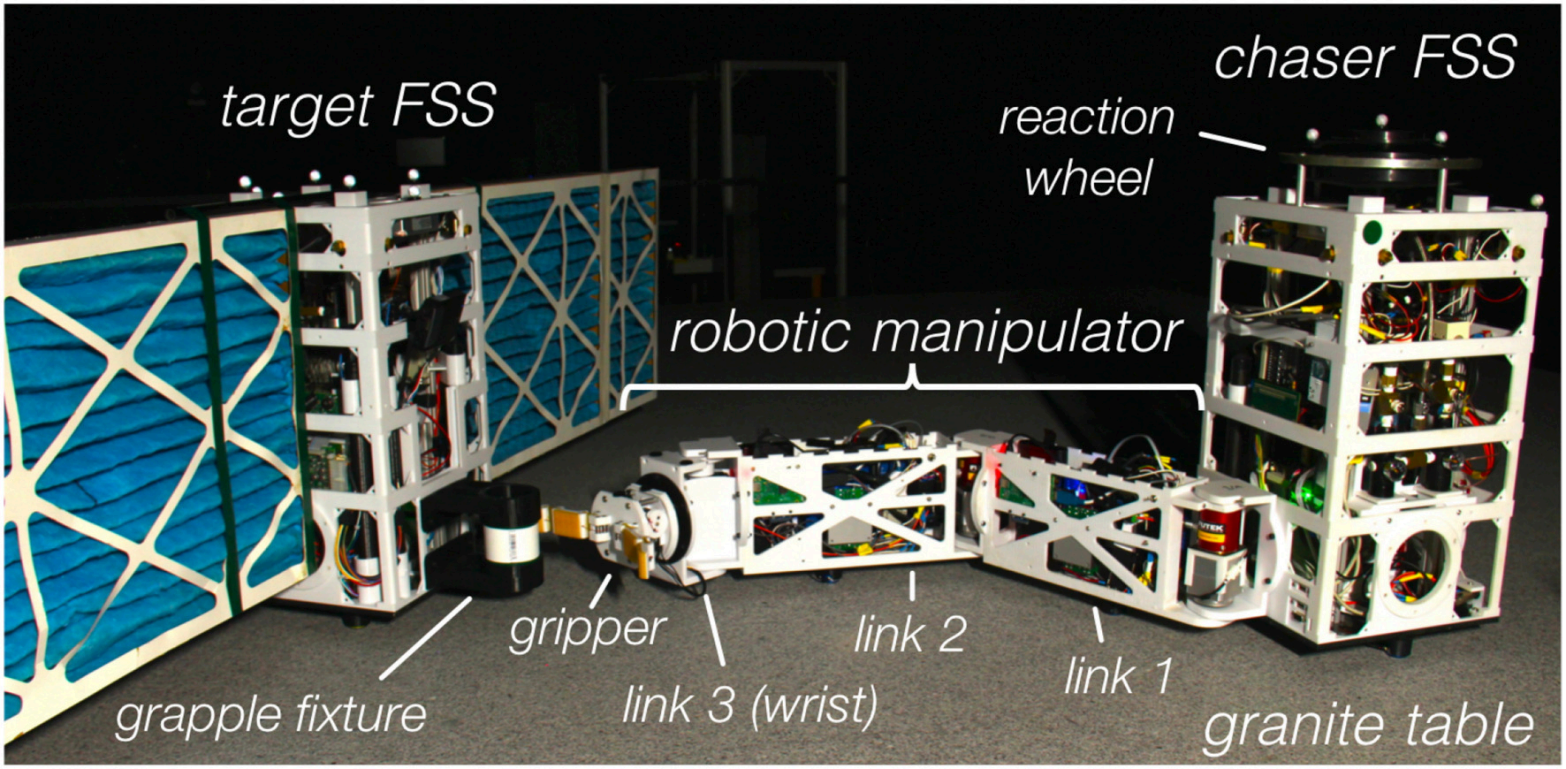
Floating Spacecraft Simulators used during the experiments at the Spacecraft Robotics Laboratory POSEIDYN planar air bearing test bed of the Naval Postgraduate School.

In order to experimentally demonstrate that the proposed maneuver actually detumbles the target upon capture and to provide empirical evidence that the proposed guidance approach is suitable for onboard implementation and real-time execution, the proposed simultaneous capture and detumble maneuver has been tested on the POSEIDYN hardware-in-the-loop dynamic planar air bearing test bed (Romano et al., 2007; Zappulla et al., 2017b). In this test bed, experimental demonstrations of docking maneuvers with tumbling objects have been previously conducted (Wilde et al., 2016; Park et al., 2017), and the set-up used in this experimental campaign, which is shown in Figure 11, is similar to the one used in previous capture-only experiments (Virgili-Llop et al., 2019).

### 6.1. Experimental Set-up

The POSEIDYN air bearing test bed consists of a smooth and horizontally leveled 4-by-4 meter granite table and multiple Floating Spacecraft Simulators (FSS) (see Zappulla et al., 2017b). A Floating Spacecraft Simulator (FSS), equipped with a three-link robotic manipulator, is used as the chaser spacecraft while a second FSS, with mock solar panels, is used as the rotating RSO to be captured.

Three planar air bearings mounted on the FSS greatly reduce its friction with the granite table. This quasi-frictionless dynamics combined with the horizontally leveled table produce a low residual acceleration environment in two translation and one rotation degree-of-freedom (planar motion). Eight cold-gas thrusters (Lugini and Romano, 2009), modulated using a Delta-Sigma modulator (Ciarcià et al., 2017; Zappulla et al., 2017a), provide the required control forces, while a reaction wheel, controlled via a speed-mode controller, actuates the requested torques (Virgili-Llop et al., 2017a). A three-link manipulator is mounted on the chaser FSS (Virgili-Llop et al., 2016, 2017a). The manipulator joints are all revolute. The first two links are identical and host an additional air bearing to support their weight. The third link of the manipulator is a minimalistic joint that functions like a wrist, allowing the gripper to adjust its orientation. The gripper is based on the open-source OpenHand Model T42 (Ma et al., 2013). Selected parameters of the FSS are provided in Table 2.

**Table 2:** Selected Floating Spacecraft Simulator parameters (Zappulla et al., 2017b).

**Parameter**	**Value**
**Floating spacecraft simulator**	
Mass	13 kg
Inertia	0.28 kg m^2^
Dimensions (length × width)	0.27 × 0.27 m
Force per thruster	~0.15 N (inlet pressure dependent)
Air tank capacity	1.868 cm^3 (14 ci)
Air tank nominal pressure	20.7 MPa (3000 psi)
Air bearings & thrusters inlet pressure	413.7 Pa (60 psi)
Onboard computer	Intel® Atom™ 1.6 GHz Z530 with 2 GB of RAM
Onoard computer performance	1900 MIPS (Dhrystone v2.1)
Real-time operating System	Linux 2.6 with the RT_PREEMPT patch (Arthur et al., 2007)
Fiber-optic rate-gyroscope	KVH® DSP-3000
Test bed residual linear acceleration	~1.871 × 10^−4^ m s-2 (or ~19.1 μg) (Zappulla et al., 2017b)
Test bed residual angular acceleration	~7.56 × 10^−2^ deg/s2 (Zappulla et al., 2017b)
**Three degree-of-freedom manipulator**	
Mass per modular link	2.9 kg
Inertia per modular link	≈0.0364 kg m^2^
Third link and gripper mass	1.128 kg
Third link and gripper inertia	≈0.012 kg m^2^
Modular link's length (axis-to-axis)	0.38 m
Third link's length (axis-to-hand)	0.18 m
Link's width	0.08 m
Motor max. torque	±1.8 N m (2.5 N m for the third joint)
Encoder resolution	150′′ (317′′ for the third joint)
Max. joint angular displacement	± 90°
Gripper	OpenHand Model T42 (flexure-flexure) (Ma et al., 2013)
Control and telemetry rate	50 Hz

During the experiments, the navigation is solved by an overhead motion capture system (VICON), providing position and orientation measurements of the different FSS on the granite table. The VICON data is augmented by an onboard Fiber Optic Gyroscope (FOG) and fused with a discrete Kalman filter (Zappulla et al., 2017b).

### 6.2. Experimental Maneuver

**Figure 12:**
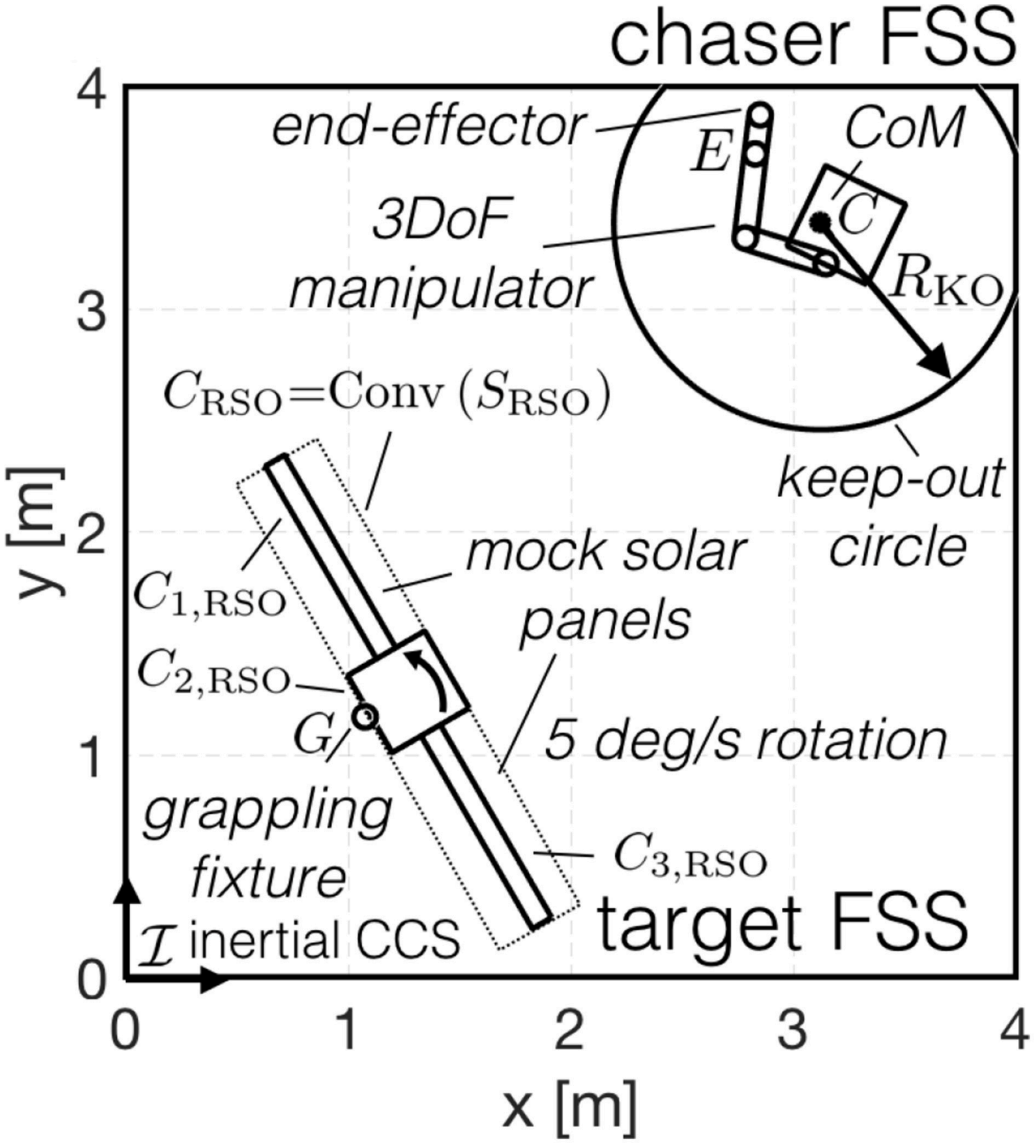
Experiment initial conditions on the POSEIDYN test bed.

The nominal initial conditions of the maneuver are shown in Figure 12. These initial conditions present a non-trivial guidance problem, requiring multiple iterations on both sequential convex programming procedures. The initial conditions were selected to obtain a trajectory that remains within the 4-by-4 meter granite table. The RSO rotation rate was set at 5 deg/s (above this rate the numerical simulations show the chaser trajectory extending outside of the granite table).

During the experiments the chaser first moves to its prescribed initial position while acquiring the initial manipulator configuration. The capture maneuver starts when the RSO achieves a pre-determined initial attitude (seen in Figure 12). Then, the two-step optimization problem is solved onboard the chaser FSS every ten seconds. The solutions **τ**_0_ and $\dot{\bar{\text{u}}}$ are applied in a feed-forward arrangement with a linear-quadratic regulator in a feedback loop, correcting any deviations from the nominal trajectory (Virgili-Llop et al., 2017a). The open-source nonlinear programming solver IPOPT (Wächter and Biegler, 2005) is used to solve the convex programming problems onboard the chaser FSS and the open-source SPART toolkit (Virgili-Llop, 2017) is used to obtain all the manipulator-related kinematic and dynamic quantities. Selected guidance parameters are provided in Table 3.

**Table 3:** Selected guidance parameters used during the experiments.

**. Parameter**	**Value**
**Guidance Algorithm**	
Guidance re-compute rate	10 s
Maneuver time *t*_*f*_	70 s
Pre-set time *t*_ps_	60 s
Number of nodes	*N*_1_ = 26 and *N*_2_ = 20
Convergence threshold	ϵ_1,2_ are set at 10% of J1,2[k−1]
End-effector error to trigger capture	≤5 cm
Post-capture manipulator deceleration period	5 s
**Chaser**	
Chaser base-spacecraft initial position	*x* = 3.3 m, *y* = 3.3 m
Manipulator initial configuration	**θ**_*m*_(*t* = 0) = [-80°,-80°,0°]
Manipulator final configuration	**θ**_*m*_(*t*_*f*_) = [-60°,60°,0°]
**RSO**	
RSO position	*x* = 1 m, *y* = 1.3 m
RSO angular velocity	5 deg/s
Nominal RSO orientation at start of maneuver	-100°
Mock solar panel dimensions	1 × 0.1 m
Main body dimensions	0.4 × 0.4 m

When the chaser reaches *t* ≥ *t*_ps_ no new guidance updates are produced and the chaser follows the latest available solution. Finally, when the manipulator's end-effector is within a set distance with respect to the grapple fixture the gripper closes, capturing the rotating RSO. The FSS emulating the RSO to be captured is also controlled up to the capture instant. Its position and orientation are controlled in order to follow the prescribed rotation rate. When the RSO is captured the control stops, allowing the combined chaser-target system to freely drift as a single rigid body.

### 6.3. Experimental Results

Ten experiments were performed sequentially in a single session with all of them successfully completing the capture and detumble maneuver. This shows that the proposed guidance is able to produce repeatable results. A video showing an example maneuver, the telemetry replays for all ten experiments, and the comparison with a capture-only maneuver is attached as Supplementary Material.

**Figure 13:**
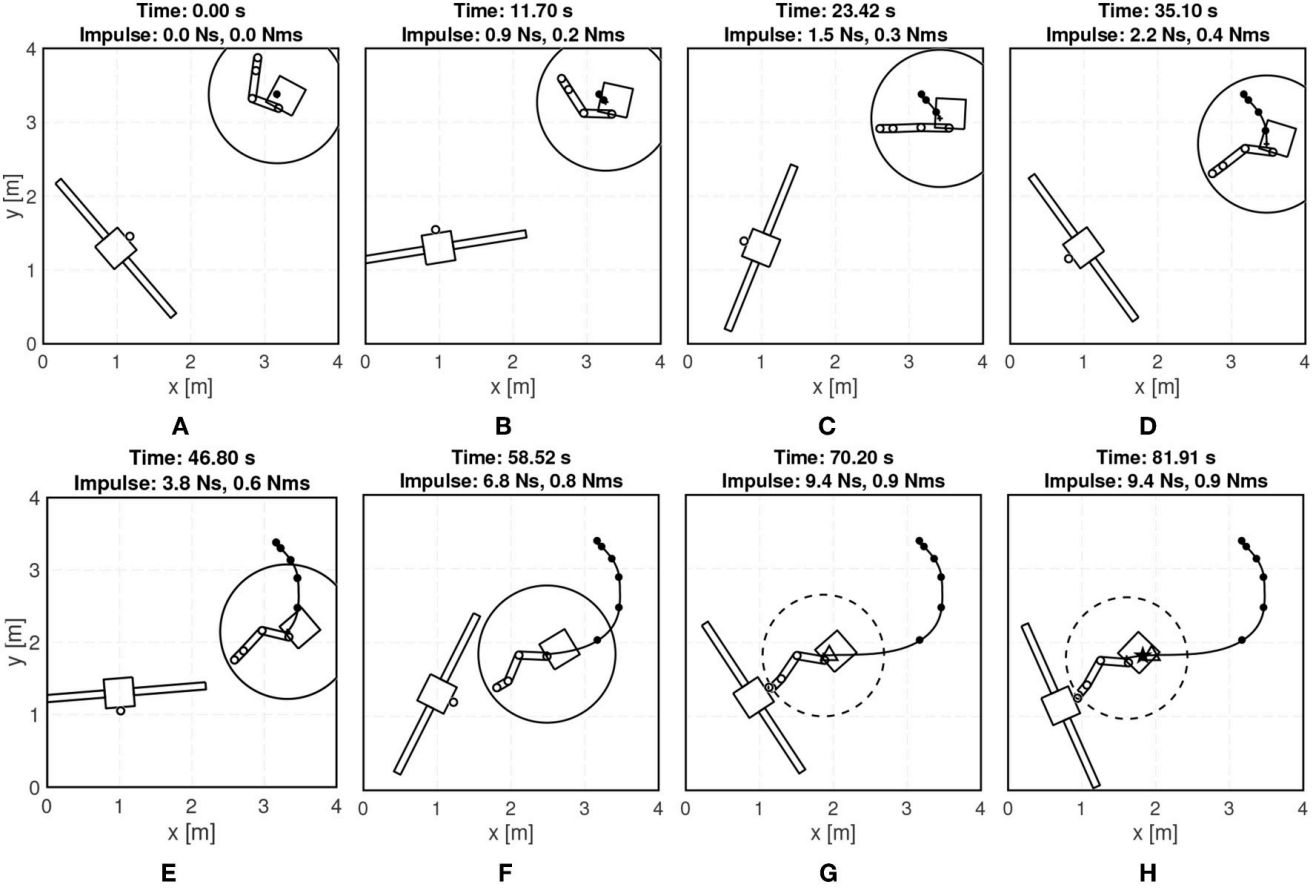
Experimental results: telemetry snapshots of the first experiment.

Eight snapshots of the first experiment are shown in Figure 13. The bold line denotes the trajectory of the chaser's center-of-mass, the bold circles, •, denote the locations where the guidance updates are delivered, the triangle, △, denotes when the gripper closes and capture achieved, and the star, ⋆, denotes when the free-drifting starts.

**Figure 14:**
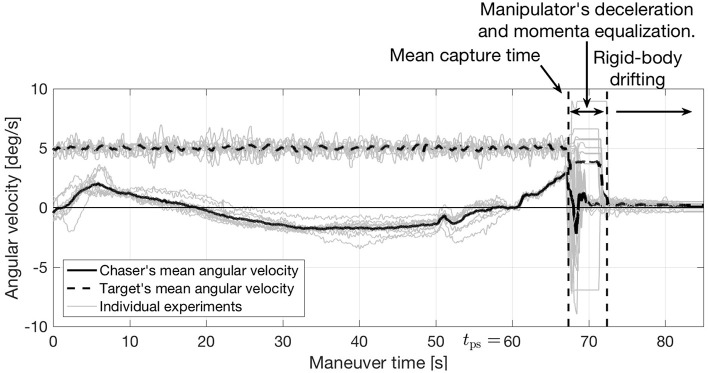
Experimental results: evolution of the angular velocity.

**Figure 15:**
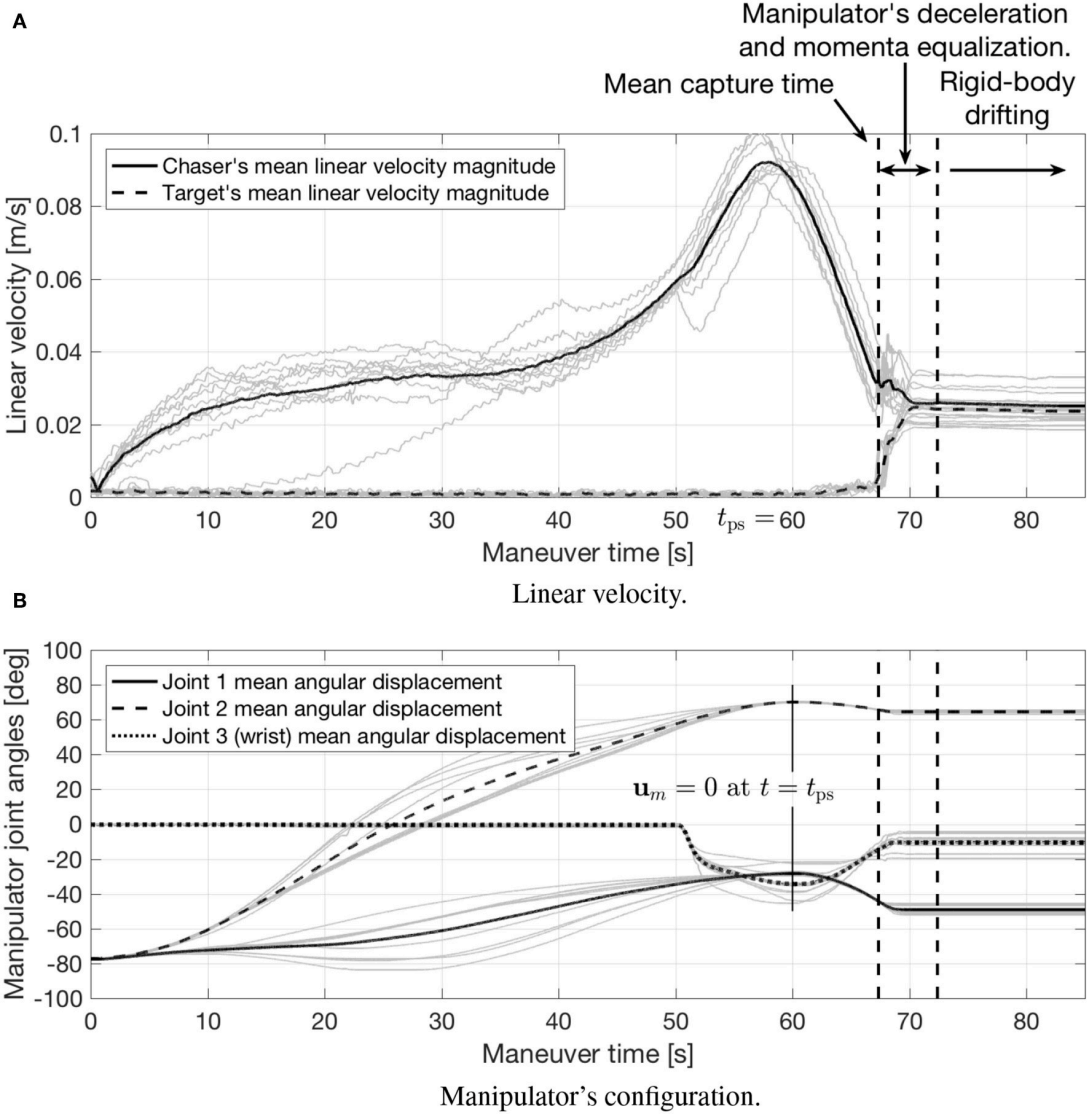
Experimental results: linear velocity and manipulator's configuration. **(A)** Linear velocity. **(B)** Manipulator's con-guration.

Figure 14 shows the evolution of the angular velocity. Additionally, Figure 15 shows how the linear velocity (Figure 15A) and manipulator's configuration (Figure 15B) evolve. Due to the accelerations imparted immediately after capture, the navigation filter on the target FSS diverges during the manipulator's deceleration period. When the forces between the chaser and target subside, the filter re-converges, revealing a chaser-target system rigidly connected with matching roto-translational states.

The detumbled rotational state achieved after the maneuver is completed is most clearly seen in Figure 14. These figures show how the angular velocity of both vehicles is reduced to a near-zero.

**Figure 16:**
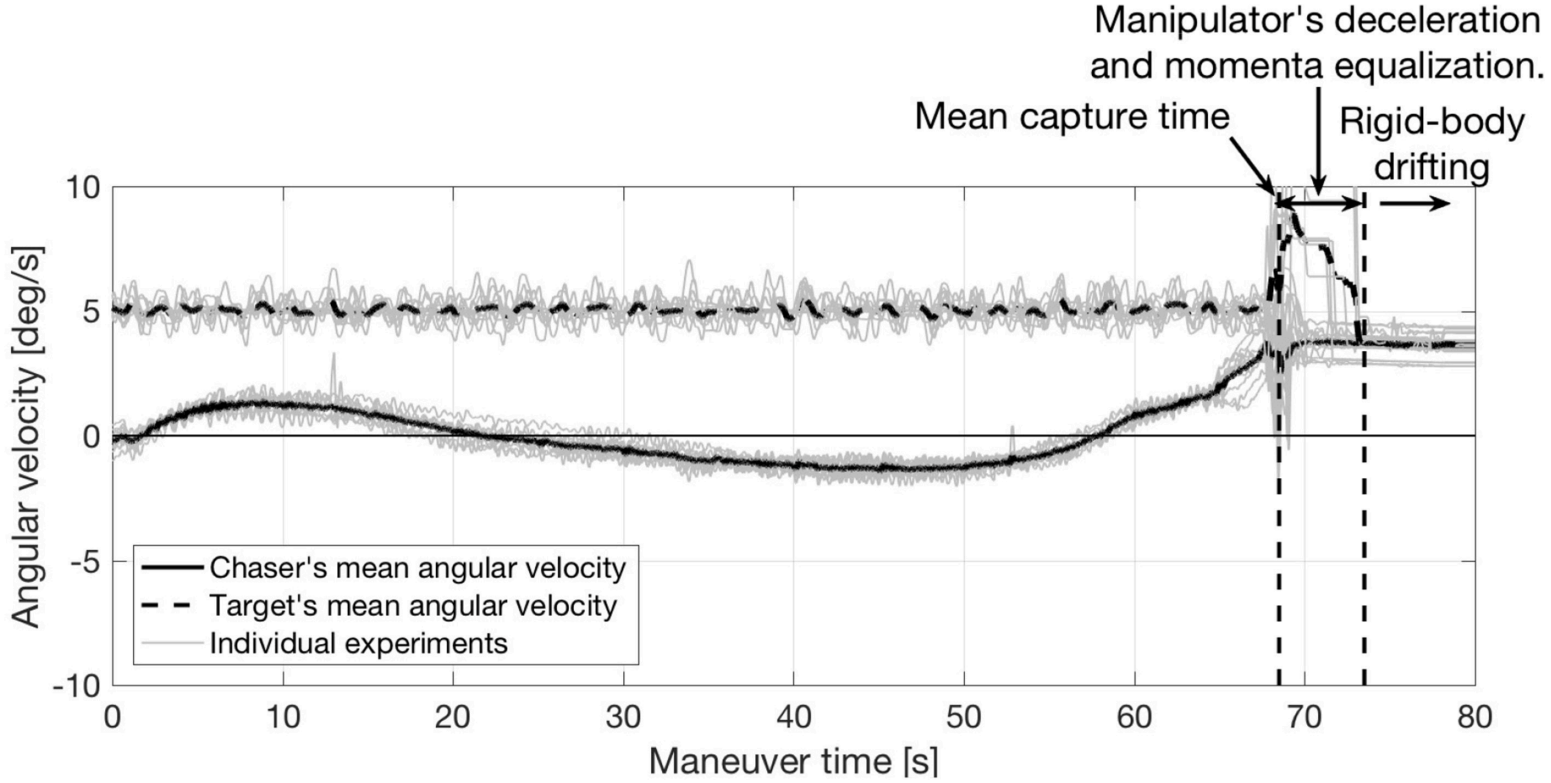
Angular velocity on capture-only maneuvers with matching velocity terminal constraints (data from Virgili-Llop et al., 2019).

To allow for a direct comparison, the time-history of the angular velocity on previously conducted capture-only maneuvers with matching velocity terminal constraints is displayed in Figure 16. As no momenta considerations are included within the maneuver's design, this figure shows how the systems retains a large angular velocity after capture. The contrast between Figure 14 (simultaneous capture and detumble) and Figure 16 (capture-only) is a testament to the simultaneous capture and detumble maneuver's effectiveness.

Going back to the results of the proposed maneuver, Figure 15A shows the linear velocity magnitude of both vehicles, and how their linear velocities converge after the momenta equalization is complete to a non-zero value, as anticipated in section 3.3. The post-capture manipulator deceleration, equalizing the momenta between both vehicles, is shown in Figure 15B. The condition ***u***_*m*_ = **0** imposed at *t* = *t*_ps_ (see section 3.2) can be also seen in this figure.

**Figure 17:**
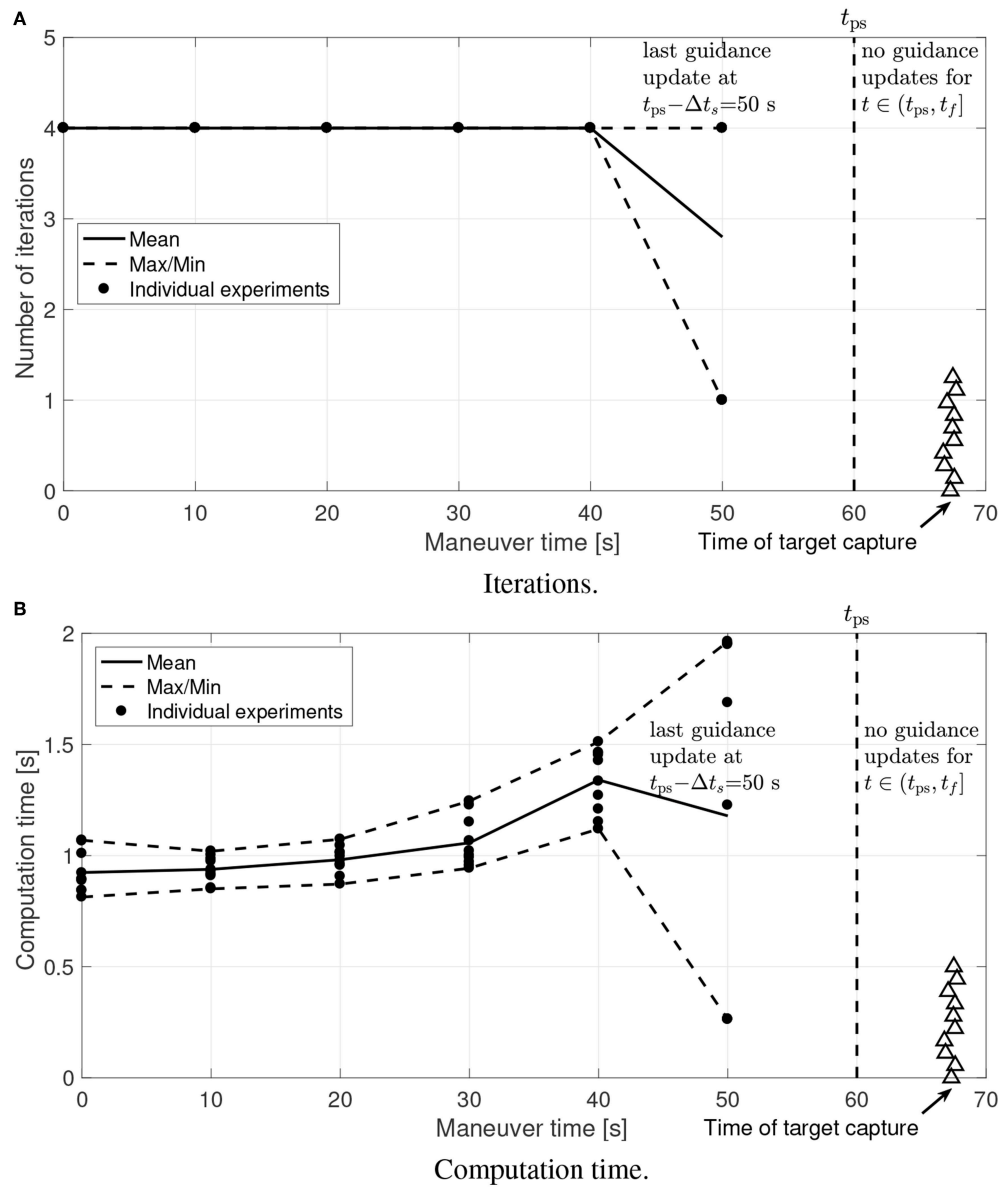
Experimental results: Step 1 computational effort. **(A)** Iterations. **(B)** Computation time.

**Figure 18:**
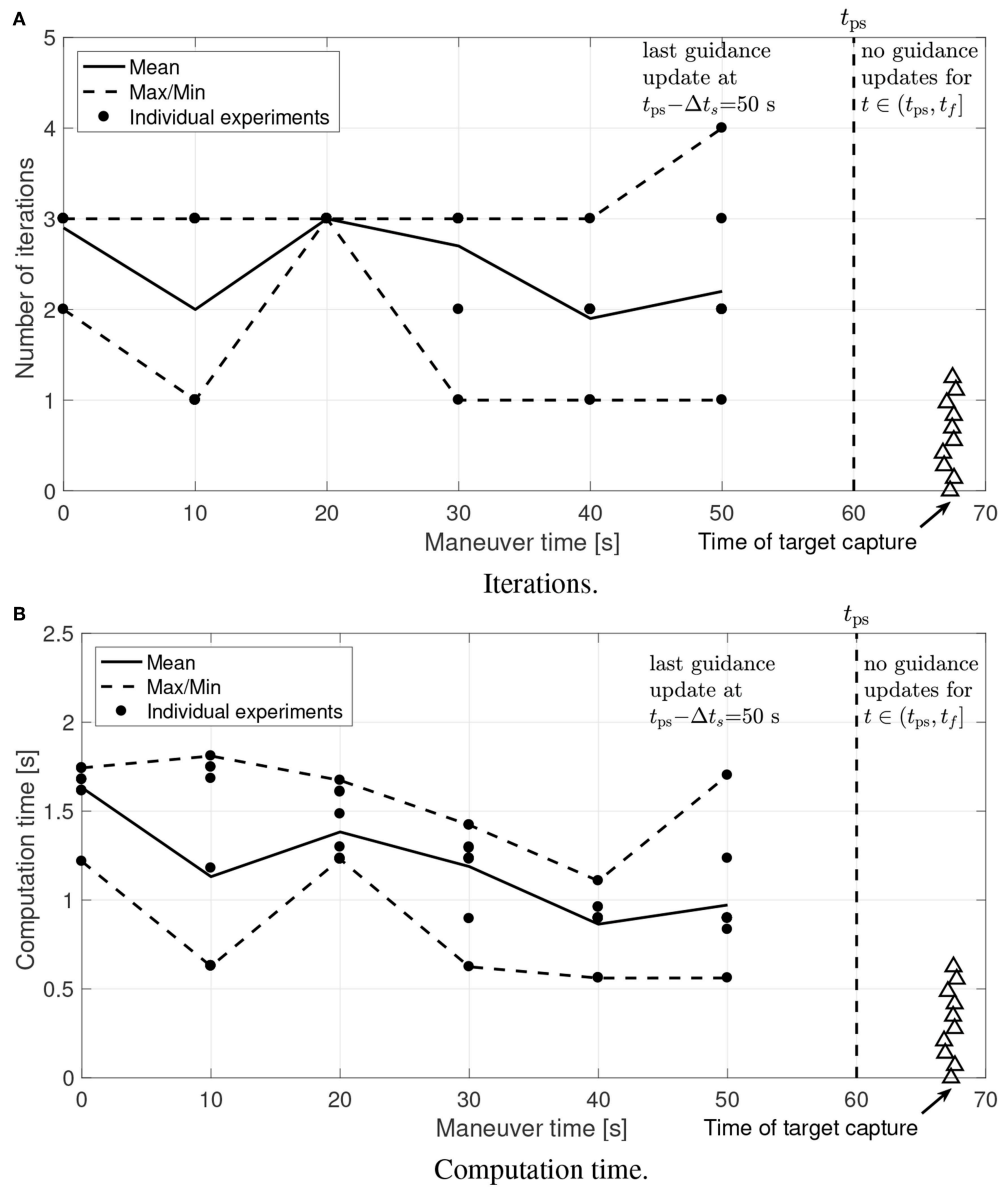
Experimental results: Step 2 computational effort. **(A)** Iterations. **(B)** Computation time.

Figures 17, 18 show the number of iterations required to converge and the total amount of computational time used on both optimization steps. It is worth pointing out that the number of iterations required to converge is small and the required computation time is well within the limits. These results confirm that the proposed guidance is suitable for onboard implementation and real-time use.

The experiments success also indicate that the proposed guidance is able to cope with the inherent uncertainties and variability present in hardware-in-the-loop experiments. During the experiments, the target uses its thrusters to keep itself at the nominal position and rotating at the specified rate. The target's control is effective but position and angular rate fluctuations are still present (as clearly seen in Figure 14). Additionally, the discrete actuation of the chaser's thrusters along with inertia uncertainties and unmodeled hardware effects deviate the chaser from its intended trajectory. Periodically recomputing the guidance trajectory allows to adjust for both chaser and target off-nominal positions, while the tracking controller helps the chaser stick to the reference trajectory.

## 7. Conclusions

A maneuver to simultaneous capture and detumble a space object has been proposed here for the first time. In order to detumble the target upon capture, a terminal constraint on the chaser's momenta is introduced. A solution to the guidance problem is obtained by solving a collection of convex programming problems. Given the deterministic convergence properties of convex programming, the proposed guidance algorithm is suitable for onboard implementation and real-time use. Numerical simulations revealed that the feasibility of a simultaneous capture and detumble maneuver decreases as the target's angular momentum increases. The simulations also indicate that it is more difficult to obtain feasible solutions for a simultaneous capture and detumble maneuver than for a capture-only maneuver with a matching velocity terminal constraint. A set of hardware-in-the-loop experiments have provided empirical evidence of the simultaneous capture and detumble maneuver efficacy and real-time capabilities of the proposed guidance algorithm. These hardware experiments also show that the potential added difficulties of the proposed approach are not insurmountable, at least for moderate rotation rates and when the target RSO is of similar size than the chaser spacecraft. Larger RSO or higher rates may impose more stringent requirements on the chaser-spacecraft and manipulator design, potentially limiting its applicability.

## Author Contributions

JV-L and MR developed the simultaneous capture and detumble maneuver and its associated guidance. Additionally JV-L performed the numerical simulations and experiments.

### Conflict of Interest Statement

The authors declare that the research was conducted in the absence of any commercial or financial relationships that could be construed as a potential conflict of interest.
